# Management of critically ill patients with COVID-19 in ICU: statement from front-line intensive care experts in Wuhan, China

**DOI:** 10.1186/s13613-020-00689-1

**Published:** 2020-06-06

**Authors:** You Shang, Chun Pan, Xianghong Yang, Ming Zhong, Xiuling Shang, Zhixiong Wu, Zhui Yu, Wei Zhang, Qiang Zhong, Xia Zheng, Ling Sang, Li Jiang, Jiancheng Zhang, Wei Xiong, Jiao Liu, Dechang Chen

**Affiliations:** 1 Department of Critical Care Medicine, Union Hospital, Tongji Medical College, Huazhong University of Science and Technology, Wuhan, China; 2 Department of Critical Care Medicine, Zhongda Hospital, School of Medicine, Southeast University, Nanjing, China; 3 Department of Critical Care Medicine, Zhejiang Provincial People’s Hospital, Hangzhou Medical College, Hangzhou, China; 4 Department of Critical Care Medicine, Zhongshan Hospital Fudan University, Shanghai, China; 5 Department of Critical Care Medicine, Fujian Provincial Hospital, Fujian Provincial Center for Critical Care Medicine, Fuzhou, China; 6 Department of Critical Care Medicine, Huadong Hospital, Shanghai, China; 7 Department of Critical Care Medicine, Renmin Hospital, Wuhan University, Wuhan, China; 8 Emergency Department, the 900th Hospital of Joint Service Corps of Chinese PLA, Fuzhou, China; 9 Department of Critical Care Medicine, Tongji Hospital, Tongji Medical College, Huazhong University of Science and Technology, Wuhan, China; 10 Department of Critical Care Medicine, The First Affiliated Hospital of Zhejiang University, Hangzhou, China; 11 Department of Critical Care Medicine, The 1st Affiliated Hospital of GuangZhou Medical University, GuangZhou Institute of Respiratory Health, Guangzhou, China; 12 Department of Critical Care Medicine, Xuanwu Hospital, Capital Medical University, Beijing, China; 13 Department of Critical Care Medicine, Shanghai Jiaotong University, School of Medicine, Ruijin Hospital North, No. 197 Ruijin 2nd Road, Huangpu District, Shanghai, 201801 China

**Keywords:** COVID-19, Critical care, Expert statement

## Abstract

**Background:**

The ongoing coronavirus disease 2019 (COVID-2019) pandemic has swept all over the world, posing a great pressure on critical care resources due to large number of patients needing critical care. Statements from front-line experts in the field of intensive care are urgently needed.

**Methods:**

Sixteen front-line experts in China fighting against the COVID-19 epidemic in Wuhan were organized to develop an expert statement after 5 rounds of expert seminars and discussions to provide trustworthy recommendation on the management of critically ill COVID-19 patients. Each expert was assigned tasks within their field of expertise to provide draft statements and rationale. Parts of the expert statement are based on epidemiological and clinical evidence, without available scientific evidences.

**Results:**

A comprehensive document with 46 statements are presented, including protection of medical personnel, etiological treatment, diagnosis and treatment of tissue and organ functional impairment, psychological interventions, immunity therapy, nutritional support, and transportation of critically ill COVID-19 patients. Among them, 5 recommendations were strong (Grade 1), 21 were weak (Grade 2), and 20 were experts’ opinions. A strong agreement from voting participants was obtained for all recommendations.

**Conclusion:**

There are still no targeted therapies for COVID-19 patients. Dynamic monitoring and supportive treatment for the restoration of tissue vascularization and organ function are particularly important.

## Introduction

The outbreak of novel coronavirus pneumonia that was first detected in Wuhan in December 2019 resulted in a worldwide pandemic. On February 11, 2020, the World Health Organization (WHO) formally named it coronavirus disease 2019 (COVID-19). A person with laboratory confirmation of virus causing COVID-19 infection, irrespective of clinical signs and symptoms, is considered as a confirmed case [1].

Globally, more than 3,750,000 confirmed individuals and over 250,000 deaths, across more than 200 countries, territories or areas have been reported [2]. Approximately 14% of confirmed cases developed severe disease [3], while the grand fatality rate was 4.2% [2]. As the virus continues to spread at an alarming rate, healthcare workers are seeking effective and actionable management for affected patients. In China, physicians have been coping with COVID-19 for over 3 months. Most of the people who contracted COVID-19 presented with mild symptoms (80.9%), then severe (13.8%), and finally critical (4.7%) (Table 1) [4]. Most of the confirmed cases were between the ages of 30 and 70 (86.6%), diagnosed in Hubei (74.7%), with the overall fatality rate of 2.3%, and 0.3% in health workers [4]. The case fatality rate for critical cases was 49.0% [4]. Patients with underlying diseases had much higher fatality rates than patients with no underlying diseases (10.5% for cardiovascular disease, 7.3% for diabetes, 6.3% for chronic respiratory disease, 6.0% for hypertension, 5.6% for cancer, and 0.9% for none) [4]. The epidemic outbreak curve peaked around January 23–26, 2020, after which the decline ensued. A recent single-center study found that most critical patients developed organ dysfunction, where 67% were found to have acute respiratory distress syndrome (ARDS), 29% with acute kidney injury (AKI), 23% with cardiac injury, 29% with liver dysfunction, and 2% with pneumothorax [5]. Besides these epidemiological findings, Chinese experts have gained valuable experience in the management and pathology of this disease. We consider it our responsibility to share these experiences through the expert consensus.

**Table 1:** The severity of the COVID-19

Severity	Definition
Mild	No signs of pneumonia on imaging
Moderate	Fever and respiratory symptoms with radiological findings of pneumonia
Severe	Dyspnea, respiratory frequency ≥ 30/min, blood oxygen saturation ≤ 93%, PaO2/FiO2 ratio < 300, and/or lung infiltrates > 50% within 24–48 h
Critical	Respiratory failure, septic shock, and/or multiple organ dysfunction/failure.

Chinese specialists in critical care medicine were organized and worked together to develop an expert statement after five rounds of expert seminars and discussions. This statement represents a synthesis of evidence and experts’ consensus on critical care, despite the lack of clinical trials. Critical cases are characterized by exhibited respiratory failure, septic shock, and/or multiple organ dysfunction/failure [6]. In experts’ opinion, the patients should also be considered as critical cases if they are suffering from high respiratory frequency (RR ≥ 30 bpm) and low oxygen index (arterial partial pressure of oxygen (PaO_2_)/fraction of inspired oxygen (FiO_2_) ≤ 200 mmHg) under high-flow nasal cannula oxygen therapy (HFNC). The experts drew up 9 sections on the management of COVID-19 disease, mostly based on the experience in Wuhan.

## Methods

The statements were drawn up by a group of 16 front-line intensive care experts in China who fought against the COVID-19 epidemic in Wuhan. The group’s agenda was predefined. The expert group first defined clinical questions to be addressed and then designated the experts in charge of each question after a first meeting. All the questions were formulated according to the population, intervention, control, and outcome (PICO) format, which helps defining inclusion and exclusion criteria for the literature searches and identifying relevant studies. The quality of evidence was assessed using the methodology described in grades of recommendation, assessment, development, and evaluation (GRADE). The quality of evidence can be high, moderate, low, or very low. Because of the sudden outbreak of a COVID-19, the proposed question could be the subject of a recommendation as an expert opinion due to inexistent or insufficient literature. In addition, the published data on Severe Acute Respiratory Syndrome (SARS), Middle East Respiratory Syndrome (MERS) and other coronaviruses infections, as well as data on supportive care in the ICU from studies on influenza and other respiratory viral infections, ARDS and sepsis was used as indirect evidence. A total of 5 rounds of expert seminars and discussions were organized to provide trustworthy recommendation on the management of critically ill COVID-19 patients (Table 2).

**Table 2:** Statement timeline

March 15, 2020	Designating the experts in charge of each addressed question
March 19, 2020	Each expert made a detailed outline of their respective question
March 26, 2020	Discussing and resolving the problems encountered by the experts in the process of making the statements
April 2, 2020	(1) Discussing the experts’ respective statement and rational after revision; (2) first round of scoring
April 3, 2020	Guideline finalization meeting for the second round of scoring

We use the wording “we recommend”, “recommended”, “should” or “should not” for strong recommendations, “should probably”, “should probably not” or “should probably be considered” for weak recommendations, and “the experts suggest”, “the experts suggest against”, “suggested” or “not suggested” for expert opinion. The implications of the recommendation strength are presented in Table 3. The proposed recommendations were discussed one by one. At least 75% of experts agree to approve a proposal for criteria, and at least 90% of experts must agree to reach a strong agreement. In the absence of strong agreement, choose to reformulate the proposal and re-rating, in order to reach consensus. Only the expert opinions that give strong agreement are retained.

**Table 3:** Recommendations according to the GRADE methodology

	Recommendations	
Grade 1+	Strong recommendation “…we recommend…”, “…recommended…” or “…should…”	High level of evidence
Grade 2+	Weak recommendation “…should probably…” or “…should probably be considered…”	Low level of evidence
Expert opinion	Recommendation in the form of an expert opinion “…The experts suggest…”, “…suggested…”, “…The experts suggest against…”, or “…not suggested…”	Insufficient level of evidence
Grade 2-	Weak recommendation “…should probably not…”	Low level of evidence
Grade 1-	Strong recommendation “…should not…”	High level of evidence

## Areas of recommendations

The prevention and control of infections, diagnostic strategy, therapeutic management, and transportation of patients were defined. Literatures were searched via PubMed and the Cochrane Library databases. Only articles published in English or with an English abstract were included in the analysis focused on recent data according to an order of appraisal ranging from meta-analyses to randomized trials to observational research studies. The study population size and research relevance were considered for each study.

## Summary of results

According to the GRADE method and summary of the results, experts drew up 46 statements. Of these guidelines, 5 had a high level of evidence (GRADE 1 ±), 21 had a low level of evidence (GRADE 2±), and 20 were expert opinions. A strong agreement was reached for all statements after two rounds of scoring.

### I Prevention and control of infections

#### Occupational safety and health

As the front-line of the COVID-19 outbreak response, health care workers are exposed to a huge risk of infection. Therefore, health care workers must follow the standard precautionary principles and try their best to ensure the personal protection, hand hygiene, ward management, environmental ventilation, and sanitization of the object surface, so as to avoid nosocomial cross-infection.

##### Statement 1

Implementation of standard precautions, strengthening ward management, and self-management are suggested safety measures for health care workers (expert opinion).

*Rationale* Averted by the current epidemic situation of COVID-19, taking proper precautions is essential for avoiding the spread of infection among health care workers. Thus, the following points need to be considered.

As a high-risk environment, tertiary class protection is suggested for health care workers in intensive care unit (ICU). Personal protective equipment (PPE) includes disposable surgical cap, N95 mask, work uniform, disposable medical uniforms, disposable latex gloves, goggles, and full-face shields. Full-face respiratory protective devices or powered air-purifying respirators are required when performing aerosol-generating procedures. Destroying and disposing of masks properly, putting on and removing PPE, and practicing hand hygiene are necessary to avoid self-contamination. Special attention should be paid to details such as the side exposure of the eyes and wrists with glove slippage, as well as the risks of infection while removing some disposable shoe covers [7]. The hand hygiene system should be strictly implemented according to the newly developed Five Moments for Hand Hygiene included in the WHO Guidelines on Hand Hygiene in Health Care (Advanced Draft) [8].

Clinical triage system needs to be established to assess all patients at admission, allow for early recognition of possible COVID-19 cases and immediate isolation of patients with suspected disease in an area separate from other patients (source control). The number of family members and visitors who are in contact with suspected or confirmed COVID-19 patients should be limited or visiting should be prohibited altogether. The proper disposal of clinical waste should be ensured [9].

Health care workers need to self-monitor for signs of illness and self-isolate. If illness occurs, they should report it to managers and stay at home. A sensible diet, proper rest, and adequate exercise are advised to maintain physical and psychological health. Health care workers should familiarize themselves with related working procedures so as to avoid mistakes [10].

##### Statement 2

Proper ICU ward setting, necessary equipment and facilities, and strict ICU environmental disinfection, are suggested (expert opinion).

*Rationale* It is suggested to adjust measures according to the differing conditions so as to set the ICU ward rationally. Contaminated areas, potentially contaminated area and clean areas need to be strictly divided. The buffer zone should be set between every two areas. Posting eye-catching logos on each area is required to prevent straying into the wrong place. Different points of access should be set for medical staff and patients, making sure they do not get crossed. For ICU, tertiary class protection should be correctly performed in each area, which is of great importance for precaution of COVID-19 [11]. The use of negative pressure rooms with natural ventilation is recommended by the WHO guidance to prevent the spread of airborne pathogens among rooms [7, 12].

First-aid materials and medicine such as oxygen tank, electrocardiogram (ECG) monitor, defibrillator, injection pump, infusion pump, endotracheal intubation supplies, portable vacuum extractor, noninvasive ventilator, invasive ventilator, hemofiltration equipment, extracorporeal membrane oxygenation (ECMO) equipment and so on should be prepared. Other equipment, including air disinfecting machine and air cleaner, as well as medical gas systems including oxygen, compressed air, special gas, and vacuum suction systems, need to be assured too.

It is of particular importance to implement effective measures to prevent the spread of COVID-19 in ICU. Disinfection includes concomitant disinfection and terminal disinfection. Concomitant disinfection must be conducted immediately for the materials and environment contaminated by the excretion of the suspected and confirmed patients. Following the end of 1 day’s work in ICU, or the patients’ recovery or death in the isolation ward, terminal disinfection needs to be done carefully. Key disinfection objects include patients’ living supplies such as clothes and quilt, medical supplies, ground and wall space of ICU wards, the surface of desks and bed tables, as well as air [11, 13].

#### Precautions of artificial airway establishment and fiber bronchoscopy procedures in severe COVID-19 patients

Current evidence indicates that COVID-19 is mainly transmitted from person to person through droplets, contact, and even high concentrations of aerosols [6]. Large amounts of droplets and aerosol are generated by sputum suction in the airway, specimen collection, tracheal intubation, fiber bronchoscopy, tracheotomy, etc. Accordingly, surgeons are at a great risk of contamination. In order to avoid occupational exposure, recommendations during the aerosol-generating procedures in COVID-19 patients are the following:

##### Statement 3

If possible, COVID-19 patients should probably be admitted to negative pressure rooms (Grade 2+, weak recommendation).

*Rationale* Negative pressure rooms are aimed to decrease the concentration of severe acute respiratory syndrome coronavirus 2 (SARS-CoV-2) pathogens. In view of that, the risk of contamination would be decreased during the aerosol-generating procedures in such a setting. During the severe acute respiratory syndrome (SARS) epidemic, it was reported that negative pressure settings were effective in preventing cross-contamination and protecting the staff and patients inside the room [14]. According to WHO recommendations for COVID-19 patients, such locations should be with a minimum of 12 air changes per hour or at least 160 L/second/patient with natural ventilation [3].

##### Statement 4

The experts suggest that operators wear a portable air-purifying respirator with level III biosafety protection (Expert opinion).

*Rationale* An observational study reported that among 138 hospitalized patients diagnosed with confirmed COVID-19 in Zhongnan Hospital in Wuhan in January, 2020, 40 were healthcare workers [15]. Till March 15, 2020, it has been reported that over 3000 health workers were confirmed with COVID-19, among whom 14 died. The memory of what has happened during the 2003 SARS outbreak is still fresh. A systematic review showed that the healthcare workers who performed aerosol-generating procedures, including endotracheal intubation (odds ratio, 6.6), noninvasive ventilation (odds ratio, 3.1), tracheotomy (odds ratio, 4.2), and manual ventilation before intubation (odds ratio, 2.8) were at higher risk of suffering from SARS infection compared with the non-performers [16].

Most of the infections among healthcare workers occurred at the early stage of this outbreak when the self-protective directive has not yet been established and reinforced. After confirmation of human to human transmission of SARS-CoV-2, the self-protection for healthcare workers was subsequently established and reinforced from the end of January 2020. Level III biosafety protection is mandatory for intubation according to the guidance of the General Office of the National Health Committee [17].

PPE donning process should be strictly followed during high-risk operation: disposable hair cover, fit-tested N95 respirator or equivalent, fluid-resistant gown, two layers of gloves, goggle and face shield, and fluid-resistant shoe covers. The main operator should use portable air-purifying respirator. All the donning processes should be supervised by a professional nurse or assistant.

Doffing process of PPE after high-risk exposure should also be followed: hand hygiene, face shield and goggle removal, fluid-resistant gown removal, outer glove removal, shoe cover removal, inner glove removal, hand hygiene, N95 respirator or equivalent removal, and hair cover removal. The doffing process seems to be of greater importance. All the processes should also be supervised so as to reduce the risk of contamination [18].

##### Statement 5

a) The aerosol-generating operations such as tracheal intubation and tracheotomy are suggested to be performed by senior physicians or specialists in the field. An electronic laryngoscope with light emitting diode is suggested during endotracheal intubation. If possible, disposable equipment is suggested to be used. b) Fiber bronchoscopy is not suggested for patients without an artificial airway. The operation is suggested to be performed by senior physicians or professionally trained respiratory therapists. A bronchoscope with an external display is suggested for facilitating operations. If possible, the use of a disposable bronchoscope is suggested (expert opinion).

*Rationale* Large amounts of aerosols generated by incubation can increase the risk of transmission and nosocomial infection [16]. Thus, visual devices are recommended to facilitate the procedure, limit operation time [19] and ensure the distance between operator and patient. Routine fiber bronchoscopy operations are not suggested for COVID-19 patients. Meanwhile, most COVID-19 patients have few airway secretions [4] so that the indication of bronchoscopy should be strictly minimized. According to the recommendations by the Centers for Disease Control and Prevention (CDC) [20] and WHO [9], disposable medical equipment should be used for patient care if possible.

##### Statement 6

(a) Deep sedation (Richmond Agitation–Sedation Scale (RASS): 3–4) is suggested for patients during the procedure of fiber bronchoscopy. (b) The artificial airway is suggested to be connected with a three-way connector allowing access to get into the airway to perform a bronchoscopy. (c) The use of a closed airway suction device is suggested (expert opinion).

*Rationale* Severe COVID-19 patients with artificial airway tend to suffer from severe hypoxemia [15]. The patient’s secretions, droplets, and aerosols can be widely spread during the operation. Patients should be intubated within 60 s [18].

The procedure of fiber bronchoscopy should be performed gently with great caution in severe COVID-19 patients.

During bronchoscopy, following procedures should be followed to avoid aerosols spreading: artificial airway should be connected with a disposable three-way connector to a ventilator, then (a) ventilator needs to be set to standby mode, (b) the artificial airway needs to be briefly clamped, (c) the bronchoscopy should be quickly inserted into the connector, (d) the clamp should be opened, (e) ventilation should be restored [21].

For the patients requiring mechanical ventilation, it is not advisable to disconnect patients from the ventilator.

### Etiological treatment

#### Which antiviral drug can be used for the treatment of critically ill patients with COVID-19?

Even though some clinical experts insisted that antiviral therapy is unnecessary for seriously ill patients with COVID-19 since the course of disease in severe types is longer than 2 weeks, multiple virus particles have been found at the lung lesions following histopathological examination. Up to date, there is no specific antiviral drug that has been testified and globally recognized effective for treating COVID-19. In China, several antiviral drugs such as ribavirin, ganciclovir, oseltamivir, arbidol, alpha-interferon, chloroquine, lopinavir–ritonavir, and remdesivir have been used in clinical settings for the treatment of COVID-19. Among them, oseltamivir and arbidol hydrochloride are the most commonly utilized; however, these antiviral drugs were originally designed for influenza, and their efficacy and safety for COVID-19 need to be further investigated.

##### Statement 7

No antiviral drugs are proven effective and should probably be considered for SARS-CoV-2 treatment (Grade 2+, weak recommendation).

*Rationale* Ribavirin is a broad-spectrum antiviral drug. Clinical observations have suggested that early use of this drug is efficacious in containing COVID-19. To avoid possible aerosol transmission, we do not recommend alpha-interferon nebulization for COVID-19 infected patients. According to a very recently published clinical study from France, hydroxychloroquine can significantly reduce viral load in COVID-19 patients, and azithromycin can further enhance this effect [22]. In this study, combination use of hydroxychloroquine (HCQ) and azithromycin for at least 3 days at an early stage could rapidly reduce the nasopharyngeal viral load and decrease the length of hospital stay for infected patients. It should be noted that treatment with higher chloroquine diphosphate (CQ) dosage (600 mg CQ twice daily) is not recommended for severe COVID-19 due to its potential safety hazards, especially when taken concurrently with azithromycin and oseltamivir [23]. Nonetheless, a randomized controlled trial (RCT) trial conducted by Cao et al. suggested monotherapy of lopinavir-ritonavir did not bring about any clinical benefits for severe COVID-19 patients compared with standard supportive care, which may be partly caused by the higher throat viral loads in lopinavir–ritonavir group, delayed treatment initiation [24]. Of note, these clinical studies were limited by relatively small sample sizes. More large-scale and well-designed clinical trials are needed to confirm their potential therapeutic effects. Arbidol monotherapy might be better than lopinavir–ritonavir in reducing viral load in COVID-19 patients [25]. A clinical study from Gilead Sciences showed that remdesivir could improve clinical conditions in critically ill patients with COVID-19, and stop patient from receiving invasive mechanical ventilation or ECMO [26]. However, a recent multicentre study published in the *Lancet* found no benefit of remdesivir in improvement of clinical outcomes for severe COVID-19 [27]. One recent study published in N Engl J Med showed that compassionate use of remdesivir improved clinical outcomes in a subset of severe COVID-19 patients [28]. However, the absence of control groups precludes a final conclusion. The definite therapeutic effectiveness of remdesivir in the treatment of severe COVID-19 needs to be further verified. Remdesivir has been approved as a potential treatment for severe COVID-19 patients by the Japanese Ministry of Health, Labour and Welfare (MHLW) on May 7, 2020 due to the COVID-19 pandemic [29].

The main side-effects of these antivirals include QT interval elongation, bradycardia, hepatic injury, and obvious gastrointestinal reactions such as serious diarrhea and vomiting which may contributed to disease deterioration. Clinical trials testing remdisivir for the treatment of severe COVID-19 patients are underway (NCT04365725, NCT04292899).

#### How to evaluate convalescent plasma therapy in a patient with COVID-2019?

Convalescent plasma therapy belongs to passive immunization, which is used for the treatment of virus infections when specific drugs and vaccines are unavailable. Convalescent plasma, which has been used for more than one hundred years, can provide specific antibodies to neutralize and eradicate the viruses from the blood circulation. Up to date, there is no particular treatment for COVID-19. In 2014, the WHO recommended the use of convalescent plasma collected from patients who recovered from the Ebola virus infection as an empirical treatment during the outbreak [30]. During the COVID-19 epidemic period, this method was also recommended by the National Health Commission of China for the treatment of severe and critical patients [6].

##### Statement 8

Convalescent plasma therapy should probably be used for severe and critically ill patients with COVID-19 (Grade 2+, weak recommendation).

*Rationale* Convalescent plasma has been testified to suppress viremia, shorten the hospital stay, and reduce mortality during several virus epidemics. In 1918 during a Spanish influenza pandemic, convalescent plasma reduced the mortality rate by > 50% in severe patients [31]. Since then, it was also used for prophylaxis or as a treatment for several virus infections such as measles, Argentine hemorrhagic fever, influenza, chickenpox, and infection by cytomegalovirus. Over the past two decades, its efficacy and safety were confirmed during pandemics of SARS, MERS, H1N1 and H5N1 avian flu. During the SARS pandemic in 2003, eighty patients received convalescent plasma at Prince of Wales Hospital, Hong Kong. By the 22nd day, a higher discharge rate was observed in patients (*n* = 48) given convalescent plasma before day 14 than that given plasma after day 14 (58.3% vs. 15.6%; *P* < 0.001) [32]. A prospective cohort study conducted by Hung et al. showed that convalescent plasma therapy (*n* = 20) significantly reduced mortality compared to the control group (*n* = 73) (20.0% vs. 54.8%; *P* < 0.01). Meanwhile, plasma treatment lowered the upper respiratory tract virus load and decreased serum cytokines levels in patients with severe pandemic (H1N1) 2009 virus infection [33]. These studies verified the efficacy of convalescent plasma in patients with virus infections. It has been reported that among three severe MERS patients who received convalescent plasma infusion, just two showed neutralizing activity [34]. Among five critically ill patients with COVID-19 receiving mechanical ventilation convalescent plasma infusion, 3 patients were discharged, while 2 clinically ill patients improved and maintained the stable condition till the day 37 after transfusion [35]. A study performed in 10 severe COVID-19 patients found that convalescent plasma treatment could improve clinical outcomes, improve immune function, and promote absorption of lung lesions [36]. Nonetheless, just like any other treatment, convalescent plasma has its limitations. The main limitation refers to the reported studies, which are not randomized trials, but just prospective cohort studies or case series studies. Therefore, it was not possible to eliminate the influence of baseline severity and other treatments when evaluating the effects of convalescent plasma therapy. Other limitations include the risk of transmitting infections to transfusion service personnel, the need for adequate selection of donors with high neutralizing antibody titers, and the risk of other transfusion-transmitted infections [37]. However, regardless of these limitations, since there are still no specific etiological treatments for COVID-19, and convalescent plasma is available, it is reasonable to use it in the treatment of COVID-19 patients.

### Oxygen therapy and respiratory support

**Fig. 1:**
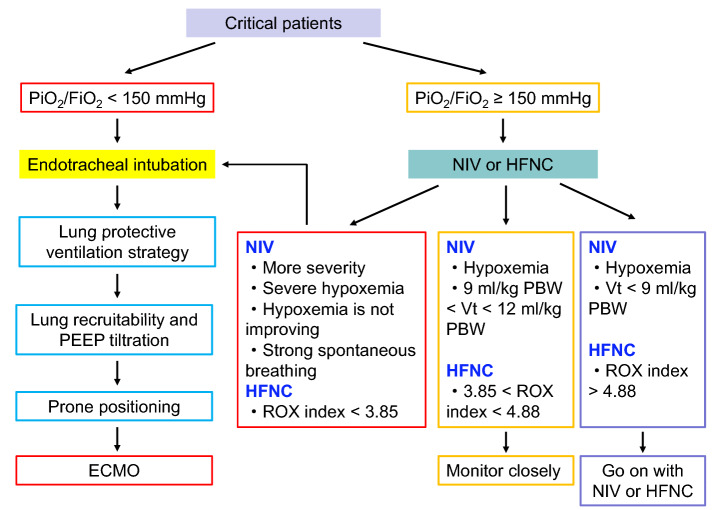
Protocol of respiratory therapy for COVID-19-induced ARDS. *NIV* non-invasive ventilation, *HFNC* high-flow nasal cannula, *PBW* predict body weight, *ECMO* extracorporeal membrane oxygenation

Respiratory failure is the primary organ dysfunction, which worsens the prognosis of COVID-19 patients. Oxygen therapy and respiratory support are the key treatments for COVID-19-induced ARDS. Due to inflammatory and necrosis-induced small airway occlusion, which was confirmed by autopsy of COVID-19-induced ARDS, positive pressure ventilation is vital to restore the collapsed airway and improve gas exchanges. However, high end-inspiratory pressure increases stress and strain to normal alveoli and increases the risk of lung injury. Oxygen therapy and respiratory support for COVID-19-induced ARDS should balance airway recruitment and risk of lung injury (Fig. 1).

#### HFNC and NIV

Indication for HFNC and NIV.

##### Statement 9

NIV and HFNC should probably be used for COVID-19-induced ARDS with PaO_2_/FiO_2_ > 150 mmHg (Grade 2+, weak recommendation).

*Rationale* Noninvasive ventilation support (NIV) and HFNC are important treatments for COVID-19-induced mild and moderate ARDS. The mechanisms of the two treatments are positive end-expiratory pressure, decreased respiratory workload, decreased incidence of intubation, ease of use, and higher comfort. In a randomized trial of adult patients admitted to the ICU for acute hypoxemic, nonhypercapnic respiratory insufficiency, continuous positive airway pressure (CPAP) delivered by face mask was associated with an early improvement in oxygenation; however, it was not associated with a reduced need for intubation or with improved outcomes [38]. A trial that compared HFNC oxygen, standard oxygen via face mask and face mask NIV in 310 patients with acute hypoxemic respiratory failure, reported that the intubation rate was significantly lower with HFNC oxygen than with standard oxygen or NIV among patients with PaO_2_/FiO_2_ ≤ 200 mmHg at enrollment and, for the whole group (patients with PaO_2_/FiO_2_ ≤ 300 mmHg), patients managed with HFNC had improved survival. There were no differences in outcomes between NIV and standard oxygen [39]. A sub-study examined the practice of NIV use in ARDS of LUNGSAFE STUDY reporting that NIV was associated with higher ICU mortality in patients with a PaO_2_/FiO_2_ < 150 mmHg [40]. For COVID-19, there is no sufficient evidence to prove that HFNC is superior to NIV.

#### Indication for intubation

##### Statement 10

When using NIV and HFNC, oxygenation and breathing patterns are suggested to be closely monitored, and intubation delays is suggested to be avoided (expert opinion).

*Rationale* For all cases with noninvasive support, patients should be closely monitored, as deterioration can abruptly occur [41]. In China, some patients presented with hypoxemia, later named “silence hypoxemia”, since these patients were without corresponding clinical manifestations, e.g., no high respiratory rates, high heart rate, respiratory distress, and other hypoxia symptoms. These patients have a high risk of sudden death and should be closely monitored and timely provided with oxygen therapy.

Positive responses are usually evident soon after the initiation of NIV and HFNC. If there is no substantial improvement in gas exchange and respiratory rate within a few hours, invasive mechanical ventilation should be started without delay. Failure to recognize a lack of improvement during noninvasive support may result in further respiratory deterioration and/or cardiac arrest, often with devastating consequences.

Delayed intubation increases ARDS mortality; therefore, early recognition of ARDS severity could avoid delayed intubation. If the use of HFNC fails, endotracheal intubation is unavoidable even with the use of rescue NIV [42].

The indications for HFNC and NIV intubation are a higher level of severity (SAPS II score > 34), hypoxemia (PaO_2_/FiO_2_ ≤ 150 mmHg), hypoxemia that is not improved following NIV treatment for 1 h, and strong spontaneous breathing (tidal volume with NIV > 12 mL/kg PBW) [43]. ROX index can be used to predict HFNC failure and intubation for patients with respiratory failure; > 4.88, suggests a high chance of success, < 3.85 suggests a high risk of failure, and intubating the patient should be discussed; index between 3.85 and 4.88, suggests the patient should be monitored very closely and intubation delays should be avoided [44].

#### Invasive ventilation

##### Statement 11

Lung-protective strategy should be used in COVID-19-induced ARDS (Grade 1+, strong recommendation).

Nine RCTs including a total of 1629 patients have compared mechanical ventilation strategies that limit tidal volumes (4–8 mL/kg predicted body weight [PBW]: males = 50 + 0.91 × [height (cm) − 152.4] kg and females = 45.5 + 0.91 × [height (cm) − 152.4] kg) and inspiratory pressures with traditional strategies (with tidal volumes 10–15 mL/kg PBW) [45, 46]. Another trial that employed a multilevel mediation analysis to analyze individual data from 3562 patients with ARDS, who were also included in nine previously reported randomized trials, identified driving pressure as the ventilation variable that best-stratified risk. Decreases in driving pressure owing to changes in ventilator settings were strongly associated with increased survival [47].

Low tidal volume (6–8 mL/kg PBW), limited plateau pressure (< 30 cmH2O), and driving pressure (< 15 cm H_2_O) could decrease ARDS mortality.

##### Statement 12

Bedside measurements should probably be used for the evaluation of lung recruitability (Grade 2+, weak recommendation).

*Rationale* Alveolar collapse is mainly generated by inflammatory lung edema, impairment of chest wall movement, and surfactant deficiency. Some reports have shown different effects of recruitment maneuvers in ARDS patients due to lung recruitability [48]. From our experience in Wuhan, most of the COVID-19 patients had low lung recruitability [49].

Due to the infectiousness of COVID-19, CT, and the other necessary equipment cannot always be used to evaluate lung recruitability. However, some bedside measurements, such as the pressure–volume curve, recruitment to inflation ratio, and clinical parameters, can be measured by a ventilator and used to evaluate lung recruitability [50].

##### Statement 13

Based on low lung recruitability in COVID-19-induced ARDS, high PEEP should probably not be used, and PEEP setting should probably be based on various factors, including gas exchange, hemodynamics, lung recruitability, and driving pressure (Grade 2+, weak recommendation).

*Rationale* Use of positive end-expiratory pressure (PEEP) usually improves gas exchange and helps reduce the need for high FiO2. In addition, appropriate levels may limit VILI by maintaining lung recruitment and improving lung homogeneity [51]. When applied with a constant Pplat, PEEP reduces the driving pressure and keeps the lung recruited.

Because of the lack of resources, PEEP selection criteria may include lung recruitability, PEEP/FiO2 table, respiratory system compliance, optimal oxygenation, and driving pressure [46, 47, 52]. Based on the available data, all PEEP values represent a compromise between the extent of recruitment and overdistension, and hemodynamics.

##### Statement 14

The experts suggest optimizing ventilator settings to improve hypercapnia (Expert opinion).

*Rationale* In China, hypercapnia has been commonly found in COVID-19-induced ARDS. The mechanisms are related to lung injury inhomogeneity and an increase in dead space. Firstly, optimization of ventilator setting is important; secondly, the prone position could decrease dead space and improve hypercapnia [53]; thirdly, tracheal gas inflation (TGI), which influences sputum drainage, could increase alveolar ventilation and CO_2_ removal [54]; fourthly, extracorporeal life support or CO_2_ removal equipment could improve hypercapnia.

#### Prone position ventilation

##### Statement 15

We recommend using prone positioning in severe COVID-19 patients to prevent the deterioration of patients’ condition (Grade 1+, strong recommendation).

*Rationale* Prone positioning has a beneficial effect on oxygenation, lung recruitment, and stress distribution. The physiological effects of prone positioning include redistribution of lung densities, often with the recruitment of well-perfused dorsal regions. Although prone positioning increases chest wall elastance, this change is usually accompanied by improved lung recruitment, a reduction in alveolar shunt and improved ventilation/perfusion ratio, subsequent improvement in oxygenation and CO_2_ clearance, a more homogeneous distribution of ventilation and a reduced VILI risk [55, 56].

Indications for prone positioning include moderate-to-severe ARDS (PaO_2_/FiO_2_ < 150 mmHg), and/or hypercapnia. Duration of prone positioning should be more than 12 h, and the termination of prone positioning should be based on the response of oxygenation, lung mechanics, and hemodynamics.

Because prone positioning could improve lung inhomogeneity, early prone positioning should be provided for COVID-19 infected patients with/without respiratory failure [57, 58] since it could prevent respiratory failure.

Since COVID-19 is highly infectious, implementation of the prone positioning might require more manpower, thus further increasing the workload of medical personnel. Pressure injury of the skin and mucous, facial edema, corneal edema, displacement of the catheter, and airway obstruction must be avoided when placing patients in the prone position.

#### Indication and timing of ECMO

Most of the COVID-19 patients presented with mild symptoms; however, about 14% of patients developed into severe cases, 5% of them were critically ill with mortality estimates of 2.3−3.83% [59–61]. Mechanical ventilation alone may not be enough to resolve refractory hypoxemia and hypercapnia in these patients. ECMO could be initiated to maintain oxygenation and avoid ventilator-induced lung injury. A cross-sectional study found that 14 (6.2%) patients treated with ECMO [62].

##### Statement 16

We recommend an early use of ECMO in COVID-19 patients with refractory hypoxemia or hypercapnia who have received invasive mechanical ventilation and prone positioning (Grade 1+, strong recommendation).

*Rationale* The appropriate timing of ECMO in COVID-19 patients might be challenging due to enormous demand and uncertainty related to the reversibility of impaired lungs. To guarantee the reversibility of compromised lungs, ECMO should be launched before injurious mechanical ventilation, which is common in critically ill patients with COVID-19 [63, 64]. The primary purpose of ECMO is the maintenance of sufficient oxygenation, removal of CO_2_, avoidance of high respiratory drive, and sequencing of ventilator-induced lung injury. The following traditional indications for ECMO may be suitable for COVID-19 patients: PaO_2_/FiO_2_ < 50 for over 3 h; PaO_2_/FiO_2_ < 80 for over 6 h; Irreversible pH < 7.2 for over 6 h.

##### Statement 17

The experts suggest using the traditional indications for ECMO in hospitals with sufficient medical resources. However, for areas with poor medical resources, the indications for ECMO are suggested to be balanced between the available resources and expected outcomes (expert opinion).

*Rationale* The WHO guidance released a statement, in which they suggest referring patients with refractory hypoxemia despite lung-protective ventilation to those settings with expertise in ECMO [3]. The latest guidance document issued by ELSO also suggested that ECMO should be considered according to the standard management algorithm for ARDS in patients with viral lower respiratory tract infections [65]. However, in reality, numerous patients who met the criteria for ECMO were admitted over a short period, which was beyond the capacity of the medical resource, including workforce and equipment. In this context, the priority of the ECMO supply should be balanced between the available medical resources and disease reversibility.

Younger patients with minor or no comorbidities should be given the highest priority when resources are limited. Despite standard contradictions, patients who fit the criteria below may be excluded: (1) patients with significant comorbidities; (2) elderly patients with worsening prognosis; (3) patients on mechanical ventilation for more than 7 days.

##### Statement 18

Prone position, as well as other adjunct therapies should probably be used for critically ill patients even during ECMO (Grade 2+, weak recommendation).

*Rationale* Ventilation with the prone position, which is currently recommended by the guidelines, can improve lung heterogeneity as well as oxygenation [66]. It should be considered in the early stages of the disease rather than as a delayed attempt [58]. Prone position ventilation is currently widely applied for severe COVID-19 patients in China [67]. Even if an ultraprotective ventilation strategy is implemented with the aid of ECMO, prone ventilation is considered to benefit the recovery of the lung.

### Hemodynamic management

#### Myocardial injury in COVID-19 patients

Elevated myocardial enzymes, such as cardiac troponin T (cTnT), creatine kinase (CK), creatine kinase-MB isoenzyme (CK-MB), have been widely observed in critically ill patients with the COVID-19, indicating potential myocardial injury. A significant elevation of myocardial enzymes often indicates a poor prognosis. Most patients with elevated myocardial enzymes do not present compromised left ventricular systolic function (reduced ejection fraction) or abnormal electrocardiogram. Left ventricular diastolic dysfunction or mild-to-moderate pulmonary arterial hypertension is common in some COVID-19 patients.

##### Statement 19

Intensive hemodynamic monitoring should probably be considered for patients with hemodynamic instability. ECMO should probably be used for salvage therapy for patients with severe cardiac dysfunction (Grade 2+, weak recommendation).

*Rationale* While SARS-CoV-2 and MERS-CoV share similar pathogenicity, it has been shown that MERS-CoV can induce acute myocarditis and heart failure [68]. Elevation of biomarkers of cardiac injury is common among critically ill patients with COVID-19 and associated with a higher risk of in-hospital mortality [63, 69]. Reversible subclinical diastolic dysfunction without systolic impairment was observed in SARS [70]. Comparable to SARS, most COVID-19 patients with elevated myocardial enzymes do not present compromised left ventricular systolic function. Left ventricular diastolic dysfunction or mild-to-moderate pulmonary arterial hypertension have been commonly found in COVID-19 patients. From our experience, tachycardia such as sinus tachycardia and atrial fibrillation were also common, while compensatory tachycardia was absent, even in patients with severe hypoxia or hemodynamic collapse.

The exact mechanism of myocardial injury in COVID-19 remains unknown. It has been suggested that direct myocardial injury is mediated via angiotensin converting enzyme 2 (ACE2). ACE2-dependent myocardial infection was observed in the murine model infected with SARS-CoV [71]. One study published in N Engl J Med provides evidence that angiotensin-converting enzyme inhibitors (ACEI)/angiotensin receptor blockers (ARB) medications in COVID-19 patients did not show any association with increasing susceptibility to SARS-CoV-2 [72].

In patients with hemodynamic instability, non-invasive or invasive monitoring, such as echocardiography or thermodilution methods, should probably be used to guide fluid therapy or administration of vasoactive agents. In patients with life-threatening cardiac dysfunction, extracorporeal life support might be salvage therapy.

#### Fluid management

##### Statement 20

Hypovolemia is common in critical COVID-19 patients, easy-to-implement parameters should probably be considered for the assessment of the patient’s volumetric status (Grade 2+, weak recommendation).

*Rationale* The use of vasoactive drugs revealed that the incidence of shock in critically COVID-19 patients was 35%, and 50% in non-survivor population [5]. The shock could be the result of hypovolemia, cardiac injury, and sepsis. Fever and mouth breathing could cause large amounts of fluid loss in critical COVID-19 patients, while decreased water intake, acute gastrointestinal injury, depression, intubation, and sedation could exacerbate hypovolemia. Previous studies reported on the relationship between dehydration and mortality in severe H1N1 patients [73]. Moreover, older age, comorbidities (especially diabetes and cardiovascular disease), lower lymphocyte count, and higher D-dimer levels were identified as risk factors associated with shock [5, 74]. Cardiac injury was found in 23% critical COVID-19 patients [5], which meant poor fluid responsiveness and the risk of pulmonary edema.

For these reasons, the patients’ volumetric status, as well as the fluid responsiveness, should be dynamically assessed. One meta-analysis of 13 RCTs showed that dynamic assessment of fluid responsiveness could improve the clinically relevant outcomes in ICU, such as mortality reduction, reduced duration of ICU length of stay, and mechanical ventilation [75]. Considering the limited clinical resources in the COVID-19 pandemic, we recommend using simple bedside assessments, such as passive leg raising (PLR), lactate clearance, pulse pressure variation (PPV), and inferior vena cava (IVC) collapsibility or distensibility. A recent meta-analysis determined that the PLR induced changes in cardiac output, with a pooled sensitivity of 0.85 and a pooled specificity of 0.91 [76]. PPV also accurately predicted fluid responsiveness in critical patients. In a meta-analysis including 22 studies and 807 patients, PPV predicted fluid responsiveness with the pooled sensitivity of 0.88 and a pooled specificity of 0.89 [77]. IVC collapsibility resulted as a simple, non-invasive bedside predictor of fluid responsiveness with a sensitivity of 0.84 and a specificity of 0.90 [78]. Early lactate clearance-directed therapy was associated with reduced in-hospital mortality, shorter duration of mechanical ventilation, and shorter ICU-stay [79]. A recent observational study showed higher serum lactate levels in COVID-19 non-survivors (2.9 vs. 1.4 mm/L) [5]. Besides, additional attention should also be paid to mental states, degree of thirst, oliguria, skin temperature, and prolonged capillary refilling time as well.

##### Statement 21

Conservative fluid strategy should probably be considered for COVID-19 patients with ARDS while ensuring tissue perfusion (Grade 2+, weak recommendation).

*Rationale* Even though fluid management in COVID-19 remains unknown, it could be assumed that these patients would respond to fluid therapy in the same way as other ARDS patients. Previous studies have shown that higher cumulative fluid balance is related to the higher mortality of critically ill patients, especially in cases of ARDS [80] and/or septic shock [81]. Due to pulmonary edema in critical COVID-19 patients [82], excessive fluid therapy could increase extravascular lung water and affect gas exchange, resulting in a poor prognosis. One clinical trial found that the conservative fluid strategy improved lung function, shortened the ICU-stay length and duration of mechanical ventilation compared with a liberal strategy in patients with acute lung injury [83]. Another study reported that more than half of critically COVID-19 patients were older than 60 years [5]. When older patients develop cardiac injury and pulmonary edema, they tend to be less responsive to fluid intake [74]. Conservative fluid strategies could reduce the occurrence of positive fluid balance while ensuring tissue perfusion [83]. Although it has been reported that conservative fluid strategy and liberal strategy have a similar incidence of AKI and the requirement for renal replacement therapy (RRT) [83], it is still necessary to closely monitor the renal function of patients. At the same time, attention should be paid to maintaining electrolyte balance and acid–base balance.

##### Statement 22

(a) Crystalloids should probably be considered for fluid resuscitation in critical COVID-19 patients. (b) Albumin should probably be considered for patients with serum albumin less than 30 g/L (Grade 2+, weak recommendation).

*Rationale* To date, there are still no studies on fluid types in COVID-19 patients; thus, our observations are based on relevant studies of critically ill patients in general. A systematic review of 69 studies that included 30,020 participants revealed that using colloids (such as starches, dextrans, albumin or fresh frozen plasma, or gelatins) had no difference in mortality in critically ill patients compared to crystalloids [84]. Considering the price and accessibility, fluid resuscitation with crystalloids should probably be used for critically ill patients.

One single-center research reported that low serum albumin (36.62 ± 6.60 g/L) was associated with the progression of COVID-19 pneumonia [85], while another study found no significant differences between the non-aggravation and aggravation patients in the early stage of the disease [86]. Serum albumin level < 30 g/L was identified as an independent risk factor for the 30-day mortality in patients with community-onset pneumonia [87]. Based on the previous evidence and our clinical observations, hypoproteinemia is present in most COVID-19 patients; thus, albumin supplement should probably be used for patients with serum albumin levels below 30 g/L.

### Psychological care, sleep, pain, agitation, delirium and in severe and critical adult patients with COVID-19

#### Statement 23

Psychological and humanistic care should probably be considered for conscious patients with COVID-19 (Grade 2+, weak recommendation).

*Rationale* Besides experiencing physical impairment and stressful treatments, COVID-19 patients are being subjected to closing monitoring, and are also witnessing various events in the ward such as sudden deterioration of illness, emergency resuscitation procedures and death, all of which could lead to posttraumatic stress disorder, anxiety, and depression according to previous studies [88, 89]. It was reported that 10% to 18% of SARS survivors had symptoms related to posttraumatic stress disorder, anxiety, and depression and that emotional support, such as communication with others and sharing worries could reduce symptom severity [88]. Accordingly, psychological implications should not be ignored in coronavirus patients. Psychological health services and humanistic care could have an important role in rehabilitation. The previous study confirmed that citalopram could improve reappraisal ability and anxiety symptoms in children and adolescents [90] and that olanzapine could improve psychotic symptoms [91]. Therefore, citalopram or olanzapine should probably be used to improve the psychological symptoms in patients or intervention of the psychologists in the isolation ward who would perform psychological assessment and psychotherapy for patients with new coronary pneumonia.

#### Statement 24

The experts suggest assessing patients’ sleep quality, implementing comprehensive measures to improve sleep and reduce the incidence of delirium, thus promoting recovery (expert opinion).

#### Statement 25

Nonpharmacological strategies and pharmacotherapy, including dexmedetomidine and melatonin, should probably be considered to decrease the incidence of delirium (Grade 2+, weak recommendation).

*Rationale* Sleep abnormalities, including abnormal sleep architecture, sleep deprivation, and disruption, frequently occur in the ICU. Numerous factors can affect sleep in COVID-19 patients, such as stress, anxiety, pain, respiratory distress, tachypnea from the underlying hypoxemia, noise levels, stage lighting in the isolation ward, implementation of healthcare, procedures of healthcare workers, and the pathophysiology of the acute illness. Sleep abnormalities may not only lead to mental disorders, but could also damage tissue repair, immune regulation mechanisms and cause delirium, all of which are associated with patient’s poor prognosis [92, 93]. Nonpharmacological strategies for preventing sleep disturbances and treating delirium, such as keeping noise levels within 44 and 45 dB range (A) during the day, and less than 35 dB (A) at night [94, 95], and providing critical patients admitted to the ICU with earplugs can significantly improve patient’s sleep and reduce the risk of delirium [96]. However, in patients with sleep disturbances and delirium, pharmacotherapy care may be necessary. Medications such as dexmedetomidine [97] and melatonin [98, 99] may promote sleep and decrease the incidence of delirium, although only limited data are available in support of their use [100].

#### Statement 26

Assessing pain and preferential use of analgesia over sedation should probably be considered for COVID-19 patients (Grade 2+, weak recommendation).

*Rationale* Pain is defined as an uncomfortable physical and mental experience caused by physical injury, inflammation, or emotional stimuli. COVID-19 patients tend to experience pain due to hypoxia, long-term immobility, inflammatory storm, impairment of heart, liver, kidney, and other organ functions, procedures, and mental stress. Opioids, such as remifentanil and sufentanil, are the first-line options for analgesia in ICU according to the pain, agitation/sedation, delirium, immobility, and sleep disruption (PADIS) guidelines [101]. Sufentanil can be used for COVID-19 patients receiving invasive mechanical ventilation during the early stage of severe ARDS because of its stronger and faster onset of analgesia, and small accumulation [102]. Remifentanil is suitable for COVID-19 patients receiving invasive mechanical ventilation, especially during person–ventilator confrontation [103] due to stronger respiratory depression. Previous research has confirmed that music or relaxation may diminish anxiety and discomfort in some patients [104, 105]. Therefore, nonpharmacological pain management strategy can be used for conscious patients with COVID-19 or for patients who do not tolerate opioid therapy, such as COVID-19 patients receiving HFNC oxygen therapy or non-invasive mechanical ventilation.

Assessment of the patient’s pain is the foundation of pain management. Accordingly, a numeric rating scale (NRS) should probably be used for evaluation of pain in all COVID-19 patients able to self-report their pain. Behavioral pain scale (BPS) and critical-care pain observation tool (CPOT) should be used to evaluate pain in critically ill patients unable to express the pain for themselves. The ideal target values are: NRS < 4 points, BPS < 5 points and CPOT < 3 points.

#### Statement 27

Deep sedation should be performed for patients with severe ARDS, especially those receiving invasive mechanical ventilation, prone position, neuromuscular blockade, or ECMO treatment (Grade 1+, strong recommendation).

#### Statement 28

The experts suggest against daily sedation interruption in deeply sedated patients (Expert opinion).

*Rationale* It is well known that analgesia and sedation can eliminate pain and discomfort, reduce sympathetic nerve excitement, patient’s metabolic rate, oxygen consumption, the metabolic burden of various organs, stress, and inflammation. However, plenty of evidence suggests that deep sedation is associated with adverse outcomes, including prolonged mechanical ventilation and ICU-stay, higher mortality, lower rates of in-hospital, and 2-year follow-up survival [106–110]. Under ‘real-life’ conditions in Wuhan, deep sedation was extremely important for reducing oxygen consumption and developing tolerance to mechanical ventilation by new coronavirus patients with severe ARDS who suffered from respiratory distress, tachypnea and respiratory overdrive even after receiving invasive mechanical ventilation. Accordingly, deep sedation should be an important part of lung-protective ventilation strategy, especially during the early stage of severe ARDS. Previous studies have confirmed that daily spontaneous awakening trials (interruption of sedatives) lead to better outcomes in patients receiving mechanical ventilation [111]. However, critically ill patients with COVID-19 have a longer mechanical ventilation time, and daily sedatives interruption is not suggested for patients receiving deep sedation in order to reduce lung damage during early stage of severe ARDS.

Midazolam and propofol are the primary medications used for ICU deep sedation. The Sedation–Agitation Scale (SAS) and RASS are the reliable and valid sedation assessment tools used for assessing the depth and quality of sedation in COVID-19 patients. The SAS and RASS should be used to measure the depth after administering sedatives. The target value is RASS -3–4 points, SAS 2 points for deep sedation, and SAS 1 point. The target value of very deep sedation is RASS -5 point for patients receiving neuromuscular blocking agents [112], prone position, or ECMO treatment. We suggest a bispectral index monitoring for patients undergoing very deep sedation, if available.

#### Statement 29

Light sedation is suggested for severe COVID-19 patients receiving HFNC oxygen therapy and non-invasive mechanical ventilation, and also for critically ill patients in the recovering stage (expert opinion).

*Rationale* Agitation and anxiety, which frequently occur in COVID-19 patients, may be associated with adverse outcomes. Appropriate sedation can reduce anxiety and agitation while preserving patients’ comfort. Light sedation can maintain frequent redirection, and increase the physiologic stress response, but not increase the incidence of myocardial ischemia. We suggest the use of light sedation for COVID-19 patients receiving HFNC oxygen therapy or non-invasive mechanical ventilation. In addition, light sedation should be given to recovering patients in order to reduce the time of mechanical ventilation and the time of stay in ICU [113] when PaO_2_/FiO_2_ ≥ 150–200 mmHg.

Dexmedetomidine can be used for patients receiving light sedation due to the small respiratory depression. The target value of light sedation is SAS 3–4 points and RASS − 2 to +1 points.

### Immunity therapy

There is some evidence that immunotherapy may be effective against novel coronavirus infection. An article [114] published on the MedRixv website stated that the mortality of COVID-19 patients might be negatively related to the number of lymphocytes in patients. Both helper T cells and suppressor T cells in COVID-19 patients tend to be below normal levels and lower level of helper T cells in the severe group. The percentage of naïve helper T cells increased, and memory helper T cells decreased in severe cases. This suggested that novel coronavirus might fight the immune system; thus, early lymphocytes and T lymphoid subgroups testing are required for early intervention, which may help to avoid lymphocyte depletion.

Currently, there are several available immunomodulatory drugs, including glucocorticoid, thymosin, and immunoglobulin.

#### Glucocorticoid

##### Statement 30

Systemic corticosteroids should probably not be used for the treatment of COVID-19. For critically ill patients with ARDS at an early stage, corticosteroids should probably be prudently used at a low or moderate dose over the short course if there are no contraindications (Grade 2-, weak recommendation).

*Rationale* Glucocorticoid use in ARDS remains a controversial topic. It is well known that corticoids are beneficial in the treatment of ARDS since they can alleviate inflammatory response and delay fibrosis [115]. A retrospective study conducted in Guangzhou revealed that proper use of corticosteroids in confirmed critical SARS patients led to lower mortality and shorter hospitalization stay and was not associated with significant secondary lower respiratory infections or any other complications [116].

However, there are some inconsistencies in the existing studies. A study involving 197 patients with ARDS, showed improved oxygenation and lung injury score in less than 12 h but no change in 28-day mortality [117]. Another study found no differences in overall mortality, while mortality was increased when steroids were started after day 14 [118]. As for viral pneumonia, a few studies have found that the administration of corticosteroids in patients with influenza pneumonia is associated with increased ICU mortality [119, 120]. WHO does not recommend routine use of corticoids in the treatment of COVID-19, while treatment with methylprednisolone may be beneficial for patients who develop ARDS, as was shown by a retrospective cohort study of 201 patients with confirmed COVID-19 pneumonia admitted to Wuhan Jinyintan Hospital in China [121]. Given the inconclusive evidence and urgent clinical demand, the guidance published by China National Health Commission on March 4, 2020, suggested the use of glucocorticoids over the short time period (3 to 5 days) for patients with progressive deterioration of oxygenation indicators, rapid imaging progress, and excessive activation of inflammatory response. The dosage of methylprednisolone should not exceed 1–2 mg/kg/day. It should be noted that large doses of glucocorticoid might delay the removal of coronavirus due to immunosuppressive effects.

#### Thymosin

Thymosin is a peptide originally isolated from thymic tissue, which was initially selected for its ability to restore immune function to thymectomized mice. Thymosin may act on precursor T cells to increase the number of activated T helper cells and expression of Th1-type cytokines such as interleukin-2 and interferon-alpha. The activated DCS and Th1 cells then kill bacterial, fungal, or viral infections and lead to the stimulation of differentiation of specific B cells to antibody-producing plasma cells and an improvement in response to vaccines by stimulation of antibody production [122]. The use of thymosin alpha 1 therapy in combination with conventional medical therapies may be effective in improving clinical outcomes in a targeted population of severe sepsis [123]. Also, it has been observed that lower lymphocytes in COVID-19 patients indicate worse prognosis [114]. Thus, thymosin may theoretically have an effect on COVID-19, which needs to be further investigated.

#### Immunoglobulin

Immunoglobulin may regulate the host’s immune response in a variety of ways, but it had no effect on mortality in previous sepsis studies. At present, it is not recommended in the treatment of COVID-19.

#### Tocilizumab

A study performed in 20 severe or critical COVID-19 patients showed that tocilizumab treatment could improve clinical outcomes, promote absorption of lung lesions, improve immune function, and reduce inflammatory response [124]. However, IL-6 inhibitor sarilumab was shown to be ineffective in the treatment of severe COVID-19, leading to early termination of this clinical trial [125]. Large sample size studies using prospective cohort designs are required to verify the therapeutic effect of IL-6 inhibitors for severe COVID-19.

### Secondary infection

Great attention should be paid to secondary infection since it may worsen the patient’s prognosis. However, since the data on the epidemiology of secondary infection in COVID-19 patients are lacking, we can only make some suggestions according to our own experience and some previous studies focused on H1N1.

#### Statement 31

The experts suggest against using prophylactic antibiotics for COVID-19 patients (expert opinion).

*Rationale* Due to the nature of virus infection, it is not logical to use prophylactic antibiotics, and there is no evidence that this strategy could reduce the incidence of the secondary infection. On the other hand, according to the management guidelines of COVID-19 from WHO and China [3, 6], empiric antibiotic treatment should only be used based on the clinical diagnosis (community-acquired pneumonia, healthcare-associated pneumonia or sepsis), local epidemiology and susceptibility data, and treatment guidelines. Based on our observations from Wuhan, many severe and critical COVID-19 patients did not show any signs of bacterial infection (such as elevated WBC, PCT and similar); thus, we do not suggest the routine use of prophylactic antibiotics in COVID-19 patients, especially at the early stage or for non-intubated patients.

#### Statement 32

The experts suggest closely monitoring the signs of secondary infection, especially in critically ill patients with COVID-19 who have been admitted to ICU > 48 h (expert opinion).

*Rationale* Both long course of the disease and immunosuppressive state place the severe and critical COVID-19 patients at a high risk of secondary infection (including bacteria and fungus). Unfortunately, the data on the epidemiology of secondary infection in COVID-19 patients are lacking. However, based on the evidence from H1N1, secondary infection is very common in patients admitted to ICU > 48 h [120, 126]. Although a complete nosocomial infection prevention and control system was set up in Wuhan according to the guidelines [127, 128], ventilator-associated pneumonia and hospital acquired pneumonia were very common occurrences in the ICU. We suspect this is mainly because the medical staff is wearing heavy personal protective equipment, and heavy workload adhered to the incomplete implementation of these measures. Consequently, the strategies for nosocomial infection prevention should be effectively implemented, and multiple site samples (blood, sputum, etc.) should be routinely collected to monitor the signs of secondary infection.

### Diagnosis and treatment of COVID-19-associated coagulopathy

In clinical practice, coagulation dysfunction is commonly found in COVID-19 patients, and the symptoms range from mild disorders of coagulation indicators to disseminated intravascular coagulation (DIC). The exact etiology of COVID-19-associated coagulopathy is unclear, diverse and multifactorial, and may include direct attack by the SARS-CoV-2 on vascular endothelial cells, cytokine storm-mediated inflammation–coagulation cascades, hypoxia, and complication with sepsis. Coagulation dysfunction or thrombocytopenia is closely associated with the severity and poor prognosis in COVID-19 patients [129]. Clinicians should increase awareness of COVID-19-associated coagulopathy, which in COVID-19 patients is accompanied with the following abnormal coagulation indexes: platelet–lymphocyte ratio < 100 × 10^9^, the reduction of prothrombin time (PT) and activated partial thromboplastin time (APTT) by more than the lower limit of 99th percentile or the increase of PT by more than 3 s or APTT by more than 5 s, or the increase of fibrinogen, fibrin degradation product (FDP) and D-dimer by more than the lower limit of 99th percentile without clinical evidence of primary blood system diseases or chronic liver diseases.

#### Statement 33

Routinely assessing the coagulation dysfunction on admission and dynamically monitored thereafter should probably be performed to identify COVID-19-associated coagulopathy as early as possible (Grade 2+, weak recommendation).

*Rationale* According to the available literature, the condition of COVID-19 patients is commonly complicated with coagulopathy, where the symptoms range from mild disorders of coagulation indicators to DIC. The increase of D-dimer in COVID-19 patients is very common, accounting for 36% to 46.4% of all cases [15, 60, 64, 130, 131]. The degree of elevation and persistent elevation are indicators of poor prognosis. The Nanshan Zhong team has reported that among 1099 COVID-19 patients in 552 hospitals from 31 provinces (926 mild cases and 173 severe cases), the proportion of severely ill patients with D-123dimer higher than 0.5 mg/L was up to 59.6%, and the proportion for the mild patients was 43.2% [60]. Zhou et al. have demonstrated that among 191 confirmed COVID-19 patients (54 deaths, 171 survival), D-dimer > 1.0 g/L was an independent risk factor for clinicians to identify patients with poor prognosis at the early stage [130]. The coagulation parameters (PT and APTT) in COVID-19 patients vary with different severity and the different courses of the disease. COVID-19 patients in the early stage show the activation of the exogenous coagulation system, manifested as decreased PT and hypercoagulable state. Along with the progression of the disease, especially when patients develop DIC, PT and APTT significantly increase, which is associated with the poor prognosis of patients. Tang has reported increased fibrinogen (5.16 g/L vs. 4.51 g/L, *P* = 0.149) and FDP values (7.6 µg/mL vs. 4 µg/mL, *P* < 0.001) in COVID-19 patients [131], which indicated that instead of hyperfibrinolysis observed in the late stage of DIC, fibrinolysis inhibition is the main feature accompanying the progression of COVID-19. The autopsies of COVID-19 patients have revealed abundant transparent thrombus in the pulmonary alveoli, myocardium, portal area, and renal tubular epithelial cells, thus indicating that fibrinolysis inhibition may have a decisive role in COVID-19-associated coagulation dysfunction.

The incidence of DIC is low in COVID-19 patients. It has been reported that among the 1099 COVID-19 patients, only 1 patient (0.1%) was diagnosed as DIC [60]. However, Tang’s report has shown that the overall incidence of DIC is 8.74%. The existence of DIC was more common in fatal cases, where 71.4% met the ISTH diagnostic criteria for DIC; the median time for DIC diagnosis after admission was 4 days, whereas among the patients who survived, only 1 patient (0.6%) met this criterion [131].

Medical institutes should dynamically detect the PT, international normalized ratio (INR), APTT, D-dimer, fibrinogen, and FDP to identify COVID-19-associated coagulation disorders, which might be helpful for making timely treatment decisions. It is also suggested to use the ISTH score system to diagnose COVID-19-associated DIC [132]; if possible, SF and PAI-1 should be used to detect the pre-DIC status in the shortest possible time.

#### Statement 34

Routinely evaluating the risk of venous thromboembolism (VTE) and hemorrhage should probably be performed in COVID-19 patients. For critically ill COVID-19 patients with low hemorrhage risk, subcutaneous injection of low molecular weight heparin (LMWH) should probably be used for preventing VTE (Grade 2+, weak recommendation).

*Rationale* The most common clinical features of coagulopathy in COVID-19 patients are thrombosis in the deep vein or intermuscular vein of the lower extremity, which can be identified by the coagulation parameters and ultrasonic monitoring. It has been reported that the incidence of VTE or thrombotic complications in patients with severe COVID-19 admitted in the ICU was 25–31% [133, 134]. It is necessary to pay attention to the clinical observation of patients with bed rest lasting for more than 3 days and observe whether these patients are experiencing asymmetric pain, swelling or discomfort in unilateral lower limbs or bilateral lower limbs, or local swelling or superficial vein filling in the lateral limbs. Especially when patients show chest pain, hemoptysis, dyspnea, or hypoxemia, which cannot be explained by NCP or other basal diseases, we should be alert to the occurrence of pulmonary thromboembolism.

For critically ill COVID-19 patients with low hemorrhage risk, a subcutaneous injection of LMWH should probably be used for the prevention of VTE. For patients with severe renal dysfunction (creatinine clearance rate < 30 mL/min), unfractionated heparin is recommended. For critically ill patients whose condition is complicated with high hemorrhage risk, intermittent pneumatic compression is recommended for mechanical prevention. Mild or moderate COVID-19 patients should probably avoid sedentary lifestyle or dehydration and are encouraged to engage in active activities and to drink more water appropriately. For mild or moderate COVID-19 patients with a high or moderately high risk of VTE according to the Padua or Caprini evaluation model, it should probably be considered to use LMWH for 7 to 10 days until the elimination of risk factors.

#### Statement 35

Anticoagulation therapy should probably be used for patients with hypercoagulant state without bleeding risk. LMWH or unfractionated heparin should probably be considered to be the first choice (Grade 2+, weak recommendation).

*Rationale* Hypercoagulant state is common in COVID-19 patients. Meantime, cytokine storm-mediated inflammation–coagulation cascades may have an essential role in COVID-19-associated coagulopathy. Studies have found that in addition to the anticoagulant effect, heparin also has a certain anti-inflammatory effect [135]. Therefore, LMWH or unfractionated heparin is the first choice for anticoagulation: Tang et al. have reported that LMWH or unfractionated heparin anticoagulation was associated with improved survival in the patients with a sepsis-induced coagulopathy (SIC) score ≥ 4 and in those with D-dimer levels more than 6 times of the upper limit of normal(≥ 3 mg/L) [136]. It is suggested that LMWH 100 U/kg or unfractionated heparin 5000 units subcutaneously twice daily could be given to patients without contraindication once D-dimer ≥ 3 mg/L or SIC ≥ 4. Heparin-induced thrombocytopenia (HIT) should be prevented during heparin treatment, and platelet counting should be monitored daily. For patients with HIT, other anticoagulants, such as agatraban, bevaludine, fondaparinux, and rivaroxaban, could be used. For patients at high risk of bleeding, anticoagulants are not recommend, and Chinese traditional medicine could be used to improve blood circulation and dispersing stasis.

### Diagnosis and treatment of COVID-19-associated AKI

Although diffuse alveolar damage and ARDS are the main features of COVID-19, the involvement of the kidney and other organs needs to be considered. AKI was associated with a higher risk of in-hospital mortality. Clinicians should increase awareness of AKI in hospitalized COVID-19 patients.

#### Statement 36

Kidney disease: Improving Global Outcomes (KDIGO) criteria should probably be used for the diagnosis of AKI in COVID-19 patients. Measuring serum creatinine every 2 days should probably be performed to avoid a missed diagnosis of AKI (Grade 2+, weak recommendation).

*Rationale* The incidence of AKI in COVID-19 patients varies with different severity of illness: mild cases have an AKI incidence of 0.1–2%, severe cases have an AKI incidence of 3–3.2%, and the AKI incidence for those critical cases that require to be admitted in ICU is up to 8.3–29% [5, 15, 64, 137, 138].

According to KDIGO AKI diagnostic criteria, certifying AKI is mainly based on changes in sCr, and the frequency of sCr tests has a substantial impact on the detection rate of AKI. In a nationwide cross-sectional survey of hospitalized adult patients in China, the detection rate of AKI was only 0.99% by KDIGO criteria [139]. After adjusting for the frequency of sCr, the incidence of AKI in Chinese hospitalized adults rose to 11.6% [140]. Thus, in order to improve early recognition of AKI, sCr measurements should be performed more frequently throughout the course of the disease. It is necessary to measure sCr every 2 days throughout the course of the disease to avoid a missed diagnosis of AKI.

#### Statement 37

The experts suggest using standard AKI care bundle (5R principle) for COVID-19-associated AKI (expert opinion).

*Rationale* The exact pathogenesis of COVID-19 associated AKI is unclear. The etiology of kidney impairment in COVID-19 patients, which is likely to be diverse and multifactorial, may include direct attack by the SARS-CoV-2 on target cells in the kidney, immune system-mediated damage, disease-related prerenal factors, a complication with sepsis and nephrotoxic drug-related factors [137, 141]. COVID-19 associated AKI is an independent risk factor for poor prognosis in patients. Clinicians should address standard AKI following 5R principle (Risk screen, Recognition in the early phase, Response in time, Renal replacement therapy, and rehabilitation of the kidney). AKI is significantly more likely to develop in severe COVID-19 patients than in non-severe patients [5, 15, 64, 137, 138]. Meanwhile, studies have shown that patients with elevated baseline sCr are more likely to develop AKI and develop more severe AKI [137]. Therefore, we should routinely screen the risk of AKI in COVID -19 patients, particularly for severe cases, patients with elevated baseline sCr or those having proteinuria and hematuria at admission. Optimizing the volume status and oxygenation, maintaining hemodynamic stability, making sure the mean blood pressure above 65 mmHg are the important measures for prevention and treatment of AKI.

#### Statement 38

The experts suggest using CRRT for the critical cases accompanied by KIDGO AKI 2–3 stages, or cytokine storm syndrome (expert opinion).

*Rationale* According to the available literature [5, 15, 64, 137, 138], the percentage of COVID-19 patients who require continuous renal replacement therapy (CRRT) is 1.5–9%, and particularly the percentage of critical patients admitted in ICU that requires CRRT is 5.6–23.0%. Indications of the CRRT in COVID-19 patients include renal indications and non-renal indications. Renal indications include severe AKI (KIDGO AKI 2–3 stages) with hemodynamic instability. Non-renal indications include complications with severe ARDS and persistent inflammatory fever, which cannot be controlled not even with glucocorticoid corticosteroid therapy, hypernatremia refractory to conservative medical treatment, volume overload or urine output, which cannot meet the needs of drug infusion and energy supply and diuretic resistance.

Multiple RCT research has indicated that the application of CRRT in critical patients in an early phase cannot effectively decrease the mortality rates [142, 143]. However, considering the suggestion that restrictive fluid volume management strategy should be adopted for COVID-19 patients complicated by ARDS based on the premise of sufficient tissue perfusion, we suggest CRRT initiation in severe patients within 24 h when they show rank 2 AKI under KDIGO criteria or accompanied with cytokine storm syndrome. In clinical practice, the doctors in charge should comprehensively evaluate conditions including the COVID-19 patient’s level of systemic inflammation, severity and progress of illness, severity, and progress of AKI, local medical resources, and the qualification of blood purification operators to give a reasonable choice of CRRT application.

#### Statement 39

CRRT prescription is suggested to be target-oriented based on the patient’s condition (expert opinion).

*Rational* CRRT prescription should be prescribed before the application of CRRT on patients, and the prescription must be target-oriented. Continuous veno-venous hemofiltration (CVVH)\continuous veno-venous hemodiafiltration (CVVHDF) is the common CRRT mode to resolve severe disturbance of electrolyte and acid–base balance and correcting azotemia, while slow continuous ultrafiltration (SCUF) could be adapted for fluid overload alone.

A high proportion of critical COVID-19 patients show hypercoagulable state. Reports show that for COVID-19 patients, their APTT and PT are shortened by 16% and 30%, respectively, and 36% of patients show an increase of D-dimer concentration [131]. The autopsy pathology of COVID-19 patients displayed lots of transparent thrombus in the pulmonary alveoli, myocardium, portal area, and renal tubular epithelial cells [63]. So anticoagulation treatment with heparin should be applied with priority for patients with no or low risk of bleeding. For critical patients with active bleeding or with a high risk of bleeding, we suggest regional citrate anticoagulation (RCA) or anticoagulation without heparin. As ECMO uses systemic heparinization, no independent usage of anticoagulant is required in CRRT combined with ECMO treatment [144].

Nutritional support therapy in severe and critically ill adult patients with COVID-19

COVID-19 patients may present with fever, fatigue, and dry cough. Critical patients often develop dyspnea and/or hypoxemia after 1 week, and, later on, may develop acute respiratory distress syndrome, shock, etc. [6]. These patients are believed to suffer from malnutrition, which is linked to three different factors [145]: (1) a severe catabolic state with marked proteolysis and loss of lean body mass because of stress and inflammation. As the need for energy and protein increases, negative nitrogen balance often occurs. (2) In severe COVID-19 patients, the gastrointestinal function is impaired due to hypoxia and novel coronavirus infection. Clinically, some patients suffered from anorexia and diarrhea due to virus attack, antiviral drugs such as lopinavir/ritonavir, and anti-infective drugs. (3) Some patients receiving noninvasive mechanical ventilation are at risk of enteral nutritional intolerance and aspiration for severe abdominal distension and increased intra-abdominal pressure through ventilation. Therefore, severe and critically ill COVID-19 patients are often at high nutritional risks, particularly those with underlying diseases and the elderly or who were hospitalized in ICU for more than 48 h [145].

#### Statement 40

The experts suggest dynamically assessing the nutritional risks of COVID-19 patients and providing timely nutritional support (expert opinion).

*Rationale* One of the metabolic characteristics of COVID-19 patients is increased proteolysis and change in the amino acid spectrum. Clinical tests showed that the levels of branched-chain amino acids are decreased, and the serum pre-albumin level is often < 100 mg/L (is some cases even lower than 70 mg/L, or < 50 mg/L). NRS 2002 or the modified NUTRIC scoring tool [146] has been recommended for these patients; NRS 2002 score ≥ 3 indicates malnutrition risk, and nutrition intervention is required. For patients with high malnutrition risk who have an NRS2002 score ≥ 5 or a modified NUTRIC score ≥ 5 (without considering IL-6), nutritional therapy should be prescribed as soon as possible. For patients admitted to the ICU for more than 48 h, a nutritional risk assessment should be initiated as quickly as possible.

#### Statement 41

Early nutrition therapy within 24–48 h after admission and preferential use of enteral nutrition should probably be considered (Grade 2+, weak recommendation).

*Rationale* A meta-analyses [147] showed that a moderately hypocaloric (enteral) diet (provisioning of 50–70% of the calorie target) was superior to a severely hypocaloric diet (provisioning of about 30% of the calorie target). When analyzing the magnitude of calorie intake (severely vs. moderately hypocaloric), three meta-analyses suggested that a severely hypocaloric diet may be harmful in the acute phase.

Enteral nutrition (EN) is the preferred route of feeding for critically ill patients who require nutrition support therapy and cannot normally eat [148, 149].

Society of parenteral and enteral nutrition of China medical association has recommended five-step method to implement nutrition therapy for COVID-19 patients: elemental diet, nutrition education, oral nutritional supplement, tube feeding for EN, supplemental parenteral nutrition, parenteral nutrition (PN) and total parenteral nutrition (TPN).

Medical nutrition therapy should be initiated within the first 24 h after ICU admission in those patients who are unable to maintain sufficient volitional intake during the early acute phase of critical illness [148, 150].

Initiate EN within 24–48 h following the onset of critical illness and admission to the ICU, and increase to goals over the first week of ICU-stay.

For patients with invasive mechanical ventilation or receiving ECMO, if there is no contraindication of enteral nutrition, enteral nutrition is recommended as early as possible.

Enteral nutrition should be delayed in patients with severe COVID-19 with shock, severe hypoxia, severe acidosis, upper gastrointestinal hemorrhage or residual in stomach > 500 mL/6 h, intestinal ischemia, intestinal obstruction, and abdominal compartment syndrome.

#### Statement 42

We recommend that the targeted energy supply is 25–30 kcal/kg/day, and the targeted protein supplementation is 1.2–2.0 g/kg/day for severe patients with COVID-19 (Grade 1+, strong recommendation).

*Rationale* Part of COVID-19 patients show symptoms like diarrhea. Some severe patients with intestinal dysfunction are prone to enteral nutrition intolerance. For feeding intolerance, healthy feeding should be considered (feeding speed: 10–20 kcal/h or 10–30 mL/h). It is recommended to achieve a feeding target as 25–30 kcal/kg/d. At the same time, it is recommended that protein should be supplied by 1.5–2.0 g/kg/d (nitrogen 0.25–0.33 g/kg/d). The supply of branched-chain amino acids should be increased for promoting protein synthesis. When the protein intake is insufficient, it is recommended to add protein powder based on standard protein preparations to improve respiratory muscle function and immune function. In parenteral nutrition, the non-protein energy supply ratio: sugar/fat ratio is 50–70/50–30. Patients with severe COVID-19 and ARDS should appropriately reduce the proportion of sugar when selecting the type of enteral nutrition preparation in order to reduce the production of carbon dioxide. At the same time, because strict fluid management principles are used in these patients, it is recommended to choose high-energy-density enteral nutrition preparations in the early stage to limit excessive fluid intake.

#### Statement 43

A reasonable route of nutritional therapy should probably be chosen according to the severity of the disease and the method of respiratory support (Grade 2+, weak recommendation).

*Rationale* Severe COVID-19 patients often have trouble eating because the gastrointestinal function is impaired due to hypoxia and novel coronavirus infection. Therefore intestinal nutrition tube indwelling is should probably be used for these patients. Most of the patients with severe COVID-19 are elderly or with other comorbidities. The factors that increase the risk of aspiration are as follows: poor airway protection, aged > 70 years old, decreased level of consciousness, poor oral care, gastroesophageal reflux, and lack of nursing manpower due to the severe outbreak of COVID-19 in China. For these patients, post-pylorus feeding should be chosen. Jejuna feeding has shown to be associated with a lower rate of ventilator-associated pneumonia (< 30%) and should be delivered as a continuous infusion [149]. For those patients without a high risk of aspiration, the gastric tube should be used first because it is easy to implement.

On the other hand, for patients using non-invasive ventilation, nasal mask during feeding should probably be used to reduce the risk of hypoxemia. For those using a gastric tube, it is recommended to use the “button” mask because this type of mask is equipped with a gastric tube outlet without affecting the efficiency of ventilation. However, if patients with NIV suffer from severe abdominal distension because of severe flatulence, post-pyloric feeding should be selected. For those patients using invasive ventilation, gastric tube indwelling is recommended. However, if patients are under prone position, ventilation, feeding using a jejunal nutrient canal should be recommended.

## Transportation of COVID-19 patient

COVID-19 is a highly contagious, potentially lethal disease caused by SARS-CoV-2, especially in older patients. COVID-19 patients from non-designated hospitals, isolation sites, or fever clinics should be transferred to designated hospitals for further treatment. Critically ill patients should be transferred from general wards to ICU for critical care. COVID-19 patients should also undergo a CT scan, which requires them to move from one department to another. Thus, adequate preparation should be made to ensure the safety of patients and transport staff, as well as public health before, during, and after transportation.

### Statement 44

Before transportation, transport staff is suggested to wear a complete line of personal protective equipment, and negative pressure isolation ambulances are suggested (Expert opinion).

*Rationale* The purpose of transportation of severe COVID-19 patients to designated hospitals is to seek better medical treatment in order to improve the prognosis. The transportation of severe patients poses a great risk to other patients and hospital personnel; the risks of transportation should be balanced with benefits. When transferring suspected and confirmed cases, transport staff should take a full range of appropriate protection approaches, including wearing biohazard suits, face shields, N95 respirators, goggles, gloves, and shoe covers, and ensuring hand hygiene.

The negative pressure isolation ambulance equipped with negative air pressure and filter system should be used to make the air pressure inside the car lower than the external pressure and protect the ambiance from being contaminated. The negative pressure ambulance also needs to be equipped with mobile monitoring and transport equipment (transfer ventilator, defibrillator, etc.) and rescue medicine, and particularly an adequate supply of oxygen during transportation.

### Statement 45

During transportation, all adequate measures is suggested to be taken to ensure the safety of patients and minimum time spent on transportation (expert opinion).

*Rationale* Before transportation, the patient’s condition and necessary preparation (including dedicated route, elevator, isolated room and bed, medication, equipment, and staff) should be fully communicated to the doctors of the receiving department/hospital, and the departure time and estimated arrival time should also be informed to reduce the artificial delay in the transportation.

Before transportation, transport staff should familiarize themselves with the process of diagnosis and treatment, fully assess the overall condition of the patients, and ensure that the ambulance vehicle is in position and the transportation equipment functions well. Management of life-threatening symptoms and assessment of nasal airway patency should be performed, and an artificial airway should be established for high-risk patients in advance.

During transportation, maintenance of sufficient tissue perfusion and a relatively stable internal environment is necessary. The continuity of original monitoring and treatment should be ensured as far as possible. Invasive arterial blood pressure monitoring is suggested whenever possible. In particular, the endotracheal intubation should be prevented from displacement and falling-off, and venous access should be prevented from blockage and slippage. Patients with frequent agitation can be appropriately administered with analgesic and sedative drugs. Neuromuscular blockers could be used depending on the patient’s condition and emergency rescue capability. For ECMO patients, the body temperature, ECMO rotation speed, blood flow rate, and airflow rate should be monitored to prevent hypothermia and ECMO catheter from discounting during the transportation of an ECMO patient.

Therefore, all adequate measures should be taken to ensure the safety of patients and the minimum time spent in transportation.

### Statement 46

Transport staff is suggested to fully carry out transfer and handover, as well as post-transport decontamination (expert opinion).

*Rationale* The patient’s general condition, vital signs, specific monitoring index, and received treatment, as well as the emergency and its handling measures, should be recorded during transportation and forwarded to the receiving medical team on arrival. Medical records about patient medical history, laboratory examination results, and meaningful clinical events should be transferred to the receiving medical team.

A dedicated team should clean the dedicated route and elevator right after transport. All transport staff should wear new personal protective equipment before getting into the same ambulance for the return journey, and remove the personal protective equipment to the designated clinical area on arrival. The ambulance should be terminally cleaned after returning to the primary hospital or health care center [151]. If transport staff is exposed to infection, it should be handled in accordance with the relevant principles and practice of infectious diseases.

## Summary

Until now, the pathogenesis and etiology of COVID-19 remain unclear, and there are still no targeted therapies for COVID-19 patients except for empirically symptomatic treatments for critically ill patients. Scientists worldwide should work together and struggle to seek efficacious COVID-19 treatments.

## Data Availability

Not applicable.

## Abbreviations

ACE2: Angiotensin-converting enzyme 2
AKI: Acute kidney injury
APTT: Activated partial thromboplastin time
ARDS: Acute respiratory distress syndrome
BPS: Behavioral pain scale
CK-MB: Creatine kinase-MB isoenzyme
COVID-2019: Coronavirus disease 2019
CPAP: Continuous positive airway pressure
CPOT: Critical care pain observation tool
CQ: Chloroquine diphosphate
cTnT: Cardiac troponin T
DIC: Disseminated intravascular coagulation
ECMO: Extracorporeal membrane oxygenation
EN: Enteral nutrition
FiO_2_: Fraction of inspired oxygen
FDP: Fibrin degradation product
HCQ: Hydroxychloroquine
HFNC: High-flow nasal cannula oxygen therapy
ICU: Intensive care unit
INR: International normalized ratio
IVC: Inferior vena cava
KDIGO: Kidney Disease Improving Global Outcomes
LMWH: Low molecular weight heparin
MERS: Middle East Respiratory Syndrome
NIV: Noninvasive ventilation support
NRS: Numeric rating scale
PaO_2_: Arterial partial pressure of oxygen
PBW: Predicted body weight
PEEP: Positive end-expiratory pressure
PLR: Passive leg raising
PN: Parenteral nutrition
PPE: Personal protective equipment
PPV: Pulse pressure variation
PT: Prothrombin time
RASS: Richmond Agitation–Sedation Scale
RCT: Randomized controlled trial
SARS: Severe acute respiratory syndrome
SARS-CoV-2: Severe acute respiratory syndrome coronavirus 2
SAS: Sedation–Agitation Scale
TPN: Total parenteral nutrition
VTE: Venous thromboembolism
WHO: World Health Organization

## References

[1] World Health Organization. Global Surveillance for human infection with coronavirus disease (COVID-19). 2020. https://www.who.int/publications-detail/global-surveillance-for-human-infection-with-novel-coronavirus-(2019-ncov).

[2] World Health Organization. Coronavirus disease (COVID-2019) situation reports. 2020. https://www.who.int/emergencies/diseases/novel-coronavirus-2019/situation-reports.

[3] World Health Organization, Clinical management of severe acute respiratory infection when novel coronavirus (nCoV) infection is suspected. 2020. https://who.int/publications-detail/clinical-management-of-severe-acute-respiratory-infection-when-novel-coronavirus-(ncov)-infection-is-suspected.

[4] The Novel Coronavirus Pneumonia Emergency Response Epidemiology Team (2020). The Epidemiological Characteristics of an Outbreak of 2019 Novel Coronavirus Diseases (COVID-19)-China. China CDC Weekly.

[5] Yang X, Yu Y, Xu J, Shu H, Xia J, Liu H (2020). Clinical course and outcomes of critically ill patients with SARS-CoV-2 pneumonia in Wuhan, China: a single-centered, retrospective, observational study. Lancet Respir Med..

[6] National Health Commission & State Administration of Traditional Chinese Medicine. Diagnosis and Treatment Protocol for Novel Coronavirus Pneumonia (Trial version 7.0). 2020, Mar 3. http://www.nhc.gov.cn/xcs/zhengcwj/202003/46c9294a7dfe4cef80dc7f5912eb1989/files/ce3e6945832a438eaae415350a8ce964.pdf. 10.1097/IM9.0000000000000022 PMC7227203 40479241

[7] Wax RS, Christian MD (2020). Practical recommendations for critical care and anesthesiology teams caring for novel coronavirus (2019-nCoV) patients. Can J Anaesth.

[8] World Health Organization, Five moments for hand hygiene. https://www.who.int/gpsc/tools/Fivemoments/en/.

[9] World Health Organization, Infection prevention and control during health care when COVID-19 is suspected. 2020 March 19, https://www.who.int/publications-detail/infection-prevention-and-control-during-health-care-when-novel-coronavirus-(ncov)-infection-is-suspected-20200125.

[10] World Health Organization, Coronavirus disease (COVID-19) outbreak: rights, roles and responsibilities of health workers, including key considerations for occupational safety and health. 2020 March 19, https://www.who.int/publications-detail/coronavirus-disease-(covid-19)-outbreak-rights-roles-and-responsibilities-of-health-workers-including-keyconsiderations-for-occupational-safety-and-health.

[11] Zhe Jiang University, Handbook of COVID-19 Prevention and Treatment. 2020, http://che.zju.edu.cn/cheen/2020/0401/c27758a2021088/page.htm

[12] Alhazzani W, Møller MH, Arabi YM, Loeb M, Gong MN, Fan E (2019). Surviving Sepsis Campaign: guidelines on the management of critically ill adults with Coronavirus Disease 2019 (COVID-19). Intensive Care Med.

[13] World Health Organization, Rational use of personal protective equipment (PPE) for coronavirus disease (COVID-19). 2020 March 19 https://apps.who.int/iris/bitstream/handle/10665/331498/WHO-2019-nCoV-IPCPPE_use-2020.2-eng.pdf.

[14] Twu SJ, Chen TJ, Chen CJ, Olsen SJ, Lee LT, Fisk T (2003). Control measures for severe acute respiratory syndrome (SARS) in Taiwan. Emerg Infect Dis.

[15] Wang D, Hu B, Hu C, Zhu F, Liu X, Zhang J (2019). Clinical characteristics of 138 hospitalized patients with 2019 novel coronavirus–infected pneumonia in Wuhan, China. JAMA.

[16] Tran K, Cimon K, Severn M, Pessoa-Silva CL, Conly J (2012). Aerosol generating procedures and risk of transmission of acute respiratory infections to healthcare workers: a systematic review. PLoS ONE.

[17] General Office of National Health Committee. Guideline of Prevention and Control of Novel Coronavirus Infection Within Medical Facilities (first edition). 2020. http://www.nhc.gov.cn/yzygj/s7659/202001/b91fdab7c304431eb082d67847d27e14.shtml.

[18] Zuo MZ, Huang YG, Ma WH, Xue ZG, Zhang JQ, Gong YH (2020). Chinese Society of Anesthesiology Task Force on Airway Management: expert recommendations for tracheal intubation in critically ill patients with novel coronavirus disease 2019. Chin Med Sci J.

[19] Lewis SR, Butler AR, Parker J, Cook TM, Schofield-Robinson OJ, Smith AF (2017). Videolaryngoscopy versus direct laryngoscopy for adult patients requiring tracheal intubation: a Cochrane Systematic Review. Br J Anaesth.

[20] Centers for Disease Control and Prevention, Interim Infection Prevention and Control Recommendations for Patients with Suspected or Confirmed Coronavirus Disease 2019 (COVID-19) in Healthcare Settings, 2020. https://www.cdc.gov/coronavirus/2019-ncov/hcp/infection-control.html.

[21] Xu Y, Meng M, Li L, Liu J, Chen DC (2020). Recommendations for bronchoscopy procedures in severe COVID-19 patients. Chin J Crit Care Intensive Care Med..

[22] Gautret P, Lagier JC, Parola P, Hoang VT, Meddeb L, Sevestre J (2020). Clinical and microbiological effect of a combination of hydroxychloroquine and azithromycin in 80 COVID-19 patients with at least a six-day follow up: a pilot observational study. Travel Med Infect Dis..

[23] Borba MGS, Val FFA, Sampaio VS, Alexandre MAA, Melo GC, Brito M (2020). Effect of high vs low doses of chloroquine diphosphate as adjunctive therapy for patients hospitalized with severe acute respiratory syndrome coronavirus 2 (SARS-CoV-2) infection: a randomized clinical trial. JAMA Netw Open..

[24] Cao B, Wang Y, Wen D, Liu W, Wang J, Fan G (2020). A trial of lopinavir–ritonavir in adults hospitalized with severe Covid-19. N Engl J Med.

[25] Zhu Z, Lu Z, Xu T, Chen C, Yang G, Zha T (2020). Arbidol monotherapy is superior to lopinavir/ritonavir in treating COVID-19. J Infect.

[26] Grein J, Ohmagari N, Shin D, Diaz G, Asperges E, Castagna A (2020). Compassionate use of remdesivir for patients with severe COVID-19. N Engl J Med.

[27] Wang YM, Zhang DY, Du GH, Du RH, Zhao JP, Jin Y (2020). Remdesivir in adults with severe COVID-19: a randomised, double-blind, placebo-controlled, multicentre trial. Lancet.

[28] Grein J, Ohmagari N, Shin D, Diaz G, Asperges E, Castagna A (2020). Compassionate Use of remdesivir for patients with severe Covid-19. N Engl J Med.

[29] Gilead. Gilead Announces Approval of Veklury^®^ (remdesivir) in Japan for Patients With Severe COVID-19, 2020. https://www.gilead.com/news-and-press/press-room/press-releases/2020/5/gilead-announces-approval-of-veklury-remdesivir-in-japan-for-patients-with-severe-covid19.

[30] Casadevall A, Pirofski LA (2020). The convalescent sera option for containing COVID-19. J Clin Invest..

[31] Luke TC, Kilbane EM, Jackson JL, Hoffman SL (2006). Meta-analysis: convalescent blood products for Spanish influenza pneumonia: a future H5N1 treatment?. Ann Intern Med.

[32] Cheng Y, Wong R, Soo YOY, Wong WS, Lee CK, Ng MHL (2005). Use of convalescent plasma therapy in SARS patients in Hong Kong. Eur J Clin Microbiol Infect Dis.

[33] Hung IF, To KK, Lee CK, Lee KL, Chan K, Yan WW (2011). Convalescent plasma treatment reduced mortality in patients with severe pandemic influenza A (H1N1) 2009 virus infection. Clin Infect Dis.

[34] Ko JH, Seok H, Cho SY, Ha YE, Baek JY, Kim SH (2018). Challenges of convalescent plasma infusion therapy in Middle East respiratory coronavirus infection: a single centre experience. Antivir Ther..

[35] Shen C, Wang Z, Zhao F, Yang Y, Li J, Yuan J (2020). Treatment of 5 critically ill patients with COVID-19 with convalescent plasma. JAMA.

[36] Duan K, Liu B, Li C, Zhang H, Yu T, Qu J (2020). Effectiveness of convalescent plasma therapy in severe COVID-19 patients. Proc Natl Acad Sci USA..

[37] Marano G, Vaglio S, Pupella S, Facco G, Catalano L, Liumbruno GM (2016). Convalescent plasma: new evidence for an old therapeutic tool?. Blood Transfus..

[38] Delclaux C, L’Her E, Alberti C, Mancebo J, Abroug F, Conti G (2000). Treatment of acute hypoxemic nonhypercapnic respiratory insufficiency with continuous positive airway pressure delivered by a face mask: a randomized controlled trial. JAMA.

[39] Frat JP, Thille AW, Mercat A, Girault C, Ragot S, Perbet S (2015). High-flow oxygen through nasal cannula in acute hypoxemic respiratory failure. N Engl J Med.

[40] Bellani G, Laffey JG, Pham T, Madotto F, Fan E, Brochard L (2017). Non-invasive Ventilation of Patients with ARDS: insights from the LUNG SAFE Study. Am J Respir Crit Care Med.

[41] Agarwal R, Aggarwal AN, Gupta D (2010). Role of noninvasive ventilation in acute lung injury/acute respiratory distress syndrome: a proportion meta-analysis. Respir Care.

[42] Kangelaris KN, Ware LB, Wang CY, Janz DR, Zhuo H, Matthay MA (2016). Timing of intubation and clinical outcomes in adults with acute respiratory distress syndrome. Crit Care Med.

[43] Carteaux G, Millán-Guilarte T, De Prost N, Razazi K, Abid S, Thille AW (2016). Failure of noninvasive ventilation for de novo acute hypoxemic respiratory failure: role of tidal volume. Crit Care Med.

[44] Roca O, Caralt B, Messika J, Samper M, Sztrymf B, Hernández G (2019). An index combining respiratory rate and oxygenation to predict outcome of nasal high-flow therapy. Am J Respir Crit Care Med.

[45] Villar J, Kacmarek RM, Perez-Mendez L, Aguirre-Jaime A (2006). A high positive end-expiratory pressure, low tidal volume ventilatory strategy improves outcome in persistent acute respiratory distress syndrome: a randomized, controlled trial. Crit Care Med.

[46] Brower RG, Matthay MA, Morris A, Schoenfeld D, Thompson BT, Acute Respiratory Distress Syndrome Network (2000). Ventilation with lower tidal volumes as compared with traditional tidal volumes for acute lung injury and the acute respiratory distress syndrome. N Engl J Med.

[47] Amato MB, Meade MO, Slutsky AS, Brochard L, Costa EL, Schoenfeld DA (2015). Driving pressure and survival in the acute respiratory distress syndrome. N Engl J Med.

[48] Hodgson C, Goligher EC, Young ME, Keating JL, Holland AE, Romero L (2016). Recruitment manoeuvres for adults with acute respiratory distress syndrome receiving mechanical ventilation. Cochrane Database Syst Rev.

[49] Pan C, Chen L, Lu C, Zhang W, Xia JA, Sklar MC (2020). Lung recruitability in SARS-CoV-2 associated acute respiratory distress syndrome: a single-center, observational study. Am J Respir Crit Care Med.

[50] Chen L, Del Sorbo L, Grieco DL, Junhasavasdikul D, Rittayamai N, Soliman I (2019). Potential for lung recruitment estimated by the recruitment-to-inflation ratio in acute respiratory distress syndrome. Am J Respir Crit Care Med.

[51] Gattinoni L, Pelosi P, Crotti S, Valenza F (1995). Effects of positive end-expiratory pressure on regional distribution of tidal volume and recruitment in adult respiratory distress syndrome. Am J Respir Crit Care Med.

[52] Kacmarek RM, Villar J, Sulemanji D, Montiel R, Ferrando C, Blanco J (2016). Open lung approach for the acute respiratory distress syndrome: a pilot. Randomized controlled trial. Crit Care Med..

[53] Sud S, Friedrich JO, Taccone P, Polli F, Adhikari NK, Latini R (2010). Prone ventilation reduces mortality in patients with acute respiratory failure and severe hypoxemia: systematic review and meta-analysis. Intensive Care Med.

[54] Blanch LL (2001). Clinical studies of tracheal gas insufflation. Respir Care..

[55] Santini A, Protti A, Langer T, Comini B, Monti M, Sparacino CC (2015). Prone position ameliorates lung elastance and increases functional residual capacity independently from lung recruitment. Intensive Care Med Exp..

[56] Guerin C, Mancebo J (2015). Prone positioning and neuromuscular blocking agents are part of standard care in severe ARDS patients: yes. Intensive Care Med.

[57] Zochios V, Parhar K, Vieillard-Baron A (2018). Protecting the right ventricle in ARDS: the role of prone ventilation. J Cardiothorac Vasc Anesth.

[58] Guérin C, Reignier J, Richard JC, Beuret P, Gacouin A, Boulain T (2016). Prone positioning in severe acute respiratory distress syndrome. N Engl J Med.

[59] World Health Organization. Coronavirus disease (COVID-2019) situation reports. 2020, March 23. https://www.who.int/docs/default-source/coronaviruse/situation-reports/20200323-sitrep-63-covid-19.pdf?sfvrsn=b617302d_4.

[60] Guan WJ, Ni ZY, Hu Y, Liang WH, Ou CQ, He JX (2019). Clinical characteristics of coronavirus disease 2019 in China. N Engl J Med.

[61] Wu ZY, McGoogan JM (2019). Characteristics of and important lessons from the coronavirus disease 2019 (COVID-19) outbreak in China Summary of a report of 72314 Cases From the Chinese Center for Disease Control and Prevention. JAMA.

[62] Yu Y, Xu D, Fu S, Zhang J, Yang X, Xu L (2020). Patients with COVID-19 in 19 ICUs in Wuhan, China: a cross-sectional study. Crit Care.

[63] Huang C, Wang Y, Li X, Ren L, Zhao J, Hu Y (2020). Clinical features of patients infected with 2019 novel coronavirus in Wuhan. China. Lancet..

[64] Chen N, Zhou M, Dong X, Qu J, Gong F, Han Y (2020). Epidemiological and clinical characteristics of 99 cases of 2019 novel coronavirus pneumonia in Wuhan, China: a descriptive study. Lancet.

[65] ELSO Guidance Document: Use of ECMO in COVID-19 Patients During the Pandemic.

[66] Fan E, Del Sorbo L, Goligher EC, Hodgson CL, Munshi L, Walkey AJ (2017). An official American Thoracic Society/European Society Of Intensive Care Medicine/society of critical care medicine clinical practice guideline: mechanical ventilation in adult patients with acute respiratory distress syndrome. Am J Respir Crit Care Med.

[67] Meng L, Qiu H, Wan L, Ai Y, Xue Z, Guo Q (2020). Intubation and Ventilation amid the COVID-19 Outbreak. Anesthesiology.

[68] Alhogbani T (2016). Acute myocarditis associated with novel Middle east respiratory syndrome coronavirus. Ann Saudi Med.

[69] Shi S, Qin M, Shen B, Cai Y, Liu T, Yang F (2020). Association of cardiac injury with mortality in hospitalized patients with COVID-19 in Wuhan, China. JAMA Cardiol..

[70] Li SS, Cheng CW, Fu CL, Chan YH, Lee MP, Chan JW (2003). Left ventricular performance in patients with severe acute respiratory syndrome: a 30-day echocardiographic follow-up study. Circulation.

[71] Oudit GY, Kassiri Z, Jiang C, Liu PP, Poutanen SM, Penninger JM (2009). SARS-coronavirus modulation of myocardial ACE2 expression and inflammation in patients with SARS. Eur J Clin Invest.

[72] Mancia G, Rea F, Ludergnani M, Apolone G, Corrao G (2020). Renin-Angiotensin-Aldosterone System Blockers and the Risk of Covid-19. N Engl J Med.

[73] Fujikura Y, Kawano S, Kouzaki Y, Shinoda M, Hara Y, Shinkai M (2014). Mortality and severity evaluation by routine pneumonia prediction models among Japanese patients with 2009 pandemic influenza A (H1N1) pneumonia. Respir Investig..

[74] Ruan Q, Yang K, Wang W, Jiang L, Song J (2020). Clinical predictors of mortality due to COVID-19 based on an analysis of data of 150 patients from Wuhan, China. Intensive Care Med..

[75] Bednarczyk JM, Fridfinnson JA, Kumar A, Blanchard L, Rabbani R, Bell D (2017). Incorporating dynamic assessment of fluid responsiveness into goal-directed therapy: a systematic review and meta-analysis. Crit Care Med.

[76] Monnet X, Marik P, Teboul JL (2016). Passive leg raising for predicting fluid responsiveness: a systematic review and meta-analysis. Intensive Care Med.

[77] Yang X, Du B (2014). Does pulse pressure variation predict fluid responsiveness in critically ill patients? A systematic review and meta-analysis. Crit Care.

[78] Preau S, Bortolotti P, Colling D, Dewavrin F, Colas V, Voisin B (2017). Diagnostic accuracy of the inferior vena cava collapsibility to predict fluid responsiveness in spontaneously breathing patients with sepsis and acute circulatory failure. Crit Care Med.

[79] Pan J, Peng M, Liao C, Hu X, Wang A, Li X (2019). Relative efficacy and safety of early lactate clearance-guided therapy resuscitation in patients with sepsis: a meta-analysis. Medicine (Baltimore)..

[80] Jozwiak M, Silva S, Persichini R, Anguel N, Osman D, Richard C (2013). Extravascular lung water is an independent prognostic factor in patients with acute respiratory distress syndrome. Crit Care Med.

[81] Vincent JL, Sakr Y, Sprung CL, Ranieri VM, Reinhart K, Gerlach H (2006). Sepsis in European intensive care units: results of the SOAP study. Crit Care Med.

[82] Xu Z, Shi L, Wang Y, Zhang J, Huang L, Zhang C (2020). Pathological findings of COVID-19 associated with acute respiratory distress syndrome. Lancet Respir Med..

[83] Wiedemann HP, Wheeler AP, Bernard GR, Thompson BT, Hayden D, deBoisblanc B (2006). Comparison of two fluid-management strategies in acute lung injury. N Engl J Med.

[84] Lewis SR, Pritchard MW, Evans DJ, Butler AR, Alderson P, Smith AF (2018). Colloids versus crystalloids for fluid resuscitation in critically ill people. Cochrane Database Syst Rev.

[85] Liu W, Tao ZW, Lei W, Ming-Li Y, Kui L, Ling Z (2019). Analysis of factors associated with disease outcomes in hospitalized patients with 2019 novel coronavirus disease. Chin Med J (Engl)..

[86] Zhou Y, Zhang Z, Tian J, Xiong S (2019). Risk factors associated with disease progression in a cohort of patients infected with the 2019 novel coronavirus. Ann Palliat Med..

[87] Okumura J, Shindo Y, Takahashi K, Sano M, Sugino Y, Yagi T (2018). Mortality in patients with community-onset pneumonia at low risk of drug-resistant pathogens: impact of β-lactam plus macrolide combination therapy. Respirology.

[88] Wu KK, Chan SK, Ma TM (2005). Posttraumatic stress, anxiety, and depression in survivors of severe acute respiratory syndrome (SARS). J Trauma Stress.

[89] Lee AM, Wong JG, McAlonan GM, Cheung V, Cheung C, Sham PC (2007). Stress and psychological distress among SARS survivors 1 year after the outbreak. Can J Psychiatry.

[90] Carthy T, Benaroya-Milshtein N, Valevski A, Apter A (2017). Emotional reactivity and regulation following citalopram therapy in children and adolescents with anxiety disorders. J Child Adolesc Psychopharmacol..

[91] Flint AJ, Meyers BS, Rothschild AJ, Whyte EM, Alexopoulos GS, Rudorfer MV (2019). Effect of continuing olanzapine vs placebo on relapse among patients with psychotic depression in remission: the STOP-PD II randomized clinical trial. JAMA.

[92] Pisani MA, Friese RS, Gehlbach BK, Schwab RJ, Weinhouse GL, Jones SF (2015). Sleep in the intensive care unit. Am J Respir Crit Care Med.

[93] Boyko Y, Jennum P, Nikolic M, Holst R, Oerding H, Toft P (2017). Sleep in intensive care unit: the role of environment. J Crit Care.

[94] Akansel N, Kaymakci S (2008). Effects of intensive care unit noise on patients: a study on coronary artery bypass graft surgery patients. J Clin Nurs.

[95] Voigt LP, Reynolds K, Mehryar M, Chan WS, Kostelecky N, Pastores SM (2017). Monitoring sound and light continuously in an intensive care unit patient room: a pilot study. J Crit Care.

[96] Litton E, Carnegie V, Elliott R, Webb SA (2016). The efficacy of earplugs as a sleep hygiene strategy for reducing delirium in the ICU: a systematic review and meta-analysis. Crit Care Med.

[97] Reade MC, Eastwood GM, Bellomo R, Bailey M, Bersten A, Cheung B (2016). Effect of dexmedetomidine added to standard care on ventilator-free time in patients with agitated delirium: a randomized clinical trial. JAMA.

[98] Al-Aama T, Brymer C, Gutmanis I, Woolmore-Goodwin SM, Esbaugh J, Dasgupta M (2011). Melatonin decreases delirium in elderly patients: a randomized, placebo-controlled trial. Int J Geriatr Psychiatry..

[99] Hatta K, Kishi Y, Wada K, Takeuchi T, Odawara T, Usui C (2014). Preventive effects of ramelteon on delirium: a randomized placebo-controlled trial. JAMA Psychiatry..

[100] Fontaine GV, Der Nigoghossian C, Hamilton LA (2020). Melatonin, ramelteon, suvorexant, and dexmedetomidine to promote sleep and prevent delirium in critically ill patients: a narrative review with practical applications. Crit Care Nurs Q..

[101] Balas MC, Weinhouse GL, Denehy L, Chanques G, Rochwerg B, Misak CJ (2018). Interpreting and implementing the 2018 pain, agitation/sedation, delirium, immobility, and sleep disruption clinical practice guideline. Crit Care Med.

[102] Conti G, Arcangeli A, Antonelli M, Cavaliere F, Costa R, Simeoni F (2004). Sedation with sufentanil in patients receiving pressure support ventilation has no effects on respiration: a pilot study. Can J Anaesth.

[103] Zhu Y, Wang Y, Du B, Xi X (2017). Could remifentanil reduce duration of mechanical ventilation in comparison with other opioids for mechanically ventilated patients? A systematic review and meta-analysis. Crit Care.

[104] Cooke M, Chaboyer W, Schluter P, Foster M, Harris D, Teakle R (2010). The effect of music on discomfort experienced by intensive care unit patients during turning: a randomized cross-over study. Int J Nurs Pract..

[105] Gorji HM, Nesami BM, Ayyasi M, Ghafari R, Yazdani J (2014). Comparison of ice packs application and relaxation therapy in pain reduction during chest tube removal following cardiac surgery. N Am J Med Sci..

[106] Balzer F, Weiß B, Kumpf O, Treskatsch S, Spies C, Wernecke KD (2015). Early deep sedation is associated with decreased in-hospital and two-year follow-up survival. Crit Care.

[107] Shehabi Y, Bellomo R, Reade MC, Bailey M, Bass F, Howe B (2013). Early goal-directed sedation versus standard sedation in mechanically ventilated critically ill patients: a pilot study. Crit Care Med.

[108] Shehabi Y, Botha JA, Boyle MS, Ernest D, Freebairn RC, Jenkins IR (2008). Sedation and delirium in the intensive care unit: an Australian and New Zealand perspective. Anaesth Intensive Care.

[109] Shehabi Y, Chan L, Kadiman S, Alias A, Ismail WN, Tan MA (2013). Sedation depth and long-term mortality in mechanically ventilated critically ill adults: a prospective longitudinal multicentre cohort study. Intensive Care Med.

[110] Tanaka LM, Azevedo LC, Park M, Schettino G, Nassar AP, Réa-Neto A (2018). Early sedation and clinical outcomes of mechanically ventilated patients: a prospective multicenter cohort study. Crit Care.

[111] Girard TD, Kress JP, Fuchs BD, Thomason JW, Schweickert WD, Pun BT (2008). Efficacy and safety of a paired sedation and ventilator weaning protocol for mechanically ventilated patients in intensive care (Awakening and Breathing Controlled trial): a randomised controlled trial. Lancet.

[112] Barr J, Fraser GL, Puntillo K, Ely EW, Gélinas C, Dasta JF (2013). Clinical practice guidelines for the management of pain, agitation, and delirium in adult patients in the intensive care unit. Crit Care Med.

[113] Bucknall TK, Manias E, Presneill JJ (2008). A randomized trial of protocol-directed sedation management for mechanical ventilation in an Australian intensive care unit. Crit Care Med.

[114] Zeng Q, Li YZ, Huang G, Wu W, Dong SY, Xu Y (2020). Mortality of COVID-19 is associated with cellular immune function compared to immune function in Chinese Han population. MedRxiv..

[115] Leap J, Hill J, Patel K, Shah A (2019). Paralytics, sedation, and steroids in acute respiratory distress syndrome. Crit Care Nurs Q..

[116] Chen RC, Tang XP, Tan SY, Liang BL, Wan ZY, Fang JQ (2006). Treatment of Severe Acute Respiratory Syndrome With Glucosteroids: the Guangzhou Experience. Chest.

[117] Tongyoo S, Permpikul C, Mongkolpun W, Vattanavanit V, Udompanturak S, Kocak M (2016). Hydrocortisone treatment in early sepsis-associated acute respiratory distress syndrome: results of a randomized controlled trial. Crit Care.

[118] Steinberg KP, Hudson LD, Goodman RB, Hough CL, Lanken PN, Hyzy R (2006). Efficacy and safety of corticosteroids for persistent acute respiratory distress syndrome. N Engl J Med.

[119] Moreno G, Rodríguez A, Reyes LF, Gomez J, Sole-Violan J, Díaz E (2018). Corticosteroid treatment in critically ill patients with severe influenza pneumonia: a propensity score matching study. Intensive Care Med.

[120] Kim SH, Hong SB, Yun SC, Choi WI, Ahn JJ, Lee YJ, Lee HB, Lim CM, Koh Y (2011). Corticosteroid treatment in critically ill patients with pandemic influenza A/H1N1 2009 infection: analytic strategy using propensity scores. Am J Respir Crit Care Med.

[121] Wu C, Chen X, Cai Y, Xia J, Zhou X, Xu S (2019). Risk factors associated with acute respiratory distress syndrome and death in patients with coronavirus disease 2019 pneumonia in Wuhan, China. JAMA Intern Med.

[122] King R, Tuthill C (2016). Immune modulation with thymosin alpha 1 treatment. Vitam Horm.

[123] Wu J, Zhou L, Liu J, Ma G, Kou Q, He Z (2013). The efficacy of thymosin alpha 1 for severe sepsis (ETASS): a multicenter, single-blind, randomized and controlled trial. Crit Care.

[124] Xu X, Han M, Li T, Sun W, Wang D, Fu B (2020). Effective treatment of severe COVID-19 patients with tocilizumab. Proc Natl Acad Sci USA..

[125] U.S. Phase 2/3 adaptive-designed trial in hospitalized COVID-19 patients. https://seekingalpha.com/pr/17848770-sanofi-and-regeneron-provide-update-on-u-s-phase-2-3-adaptive-designed-trial-in-hospitalized.

[126] Schauwvlieghe AFAD, Rijnders BJA, Philips N, Verwijs R, Vanderbeke L, Van Tienen C (2018). Invasive aspergillosis in patients admitted to the intensive care unit with severe influenza: a retrospective cohort study. Lancet Respir Med..

[127] Kalil AC, Metersky ML, Klompas M, Muscedere J, Sweeney DA, Palmer LB (2016). Management of Adults With Hospital-acquired and Ventilator-associated Pneumonia: 2016 Clinical Practice Guidelines by the Infectious Diseases Society of America and the American Thoracic Society. Clin Infect Dis.

[128] Mermel LA, Allon M, Bouza E, Craven DE, Flynn P, O’Grady NP (2009). Clinical practice guidelines for the diagnosis and management of intravascular catheter-related infection: 2009 Update by the Infectious Diseases Society of America. Clin Infect Dis.

[129] Yang X, Yang Q, Wang Y, Wu Y, Xu J, Yu Y (2020). Thrombocytopenia and its association with mortality in patients with COVID-19. J Thromb Haemost.

[130] Zhou F, Yu T, Du R, Fan G, Liu Y, Liu Z (2020). Clinical course and risk factors for mortality of adult inpatients with COVID-19 in Wuhan, China: a retrospective cohort study. Lancet.

[131] Tang N, Li D, Wang X, Sun Z (2020). Abnormal coagulation parameters are associated with poor prognosis in patients with novel coronavirus pneumonia. J Thromb Haemost.

[132] Wada H, Thachil J, Di Nisio M, Mathew P, Kurosawa S, Gando S (2013). Guidance for diagnosis and treatment of DIC from harmonization of the recommendations from three guidelines. J Thromb Haemost.

[133] Cui S, Chen S, Li X, Liu S, Wang F (2020). Prevalence of venous thromboembolism in patients with severe novel coronavirus pneumonia. J Thromb Haemost.

[134] Klok FA, Kruip M, van der Meer NJM, Arbous MS, Gommers D, Kant KM, Kaptein FHJ, van Paassen J, Stals MAM, Huisman MV, Endeman H (2020). Incidence of thrombotic complications in critically ill ICU patients with COVID-19. Thromb Res.

[135] Li X, Ma X (2017). The role of heparin in sepsis: much more than just an anticoagulant. Br J Haematol.

[136] Tang N, Bai H, Chen X, Gong J, Li D, Sun Z (2019). Anticoagulant treatment is associated with decreased mortality in severe coronavirus disease 2019 patients with coagulopathy. J Thromb Haemost.

[137] Cheng Y, Luo R, Wang K, Zhang M, Wang Z, Dong L (2020). Kidney impairment is associated with in-hospital death of COVID-19 patients. MedRxiv..

[138] Guan W, Ni Z, Hu Y, Liang W, Ou C, He J (2019). Clinical characteristics of 2019 novel coronavirus infection in China. MedRxiv..

[139] Yang L, Xing G, Wang L, Wu Y, Li S, Xu G (2015). Acute kidney injury in China: a cross-sectional survey. Lancet.

[140] Xu X, Nie S, Liu Z, Chen C, Xu G, Zha Y (2015). Epidemiology and Clinical Correlates of AKI in Chinese Hospitalized Adults. Clin J Am Soc Nephrol.

[141] Yang XH, Sun RH, Chen DC (2020). Diagnosis and treatment of COVID-19: acute kidney injury cannot be ignored. Zhonghua Yi Xue Za Zhi..

[142] Gaudry S, Hajage D, Schortgen F, Martin-Lefevre L, Pons B, Boulet E (2016). Initiation strategies for renal-replacement therapy in the intensive care unit. N Engl J Med.

[143] Barbar SD, Clere-Jehl R, Bourredjem A, Hernu R, Montini F, Bruyère R (2018). Timing of Renal-Replacement Therapy in Patients with Acute Kidney Injury and Sepsis. N Engl J Med.

[144] Yang XH, Sun RH, Zhao MY, Chen EZ, Liu J, Wang HG (2020). Expert recommendation for novel coronavirus pneumonia patients with blood purification treatment. Natl Med J China..

[145] Chinese Society for Parenteral and Enteral Nutrition (2020). Recommendations for parenteral and enteral nutrition therapy in critically ill COVID-19 patients. J Surg Concept Pract..

[146] Singer P, Blaser AR, Berger MM, Alhazzani W, Calder PC, Casaer MP (2018). ESPEN guideline on clinical nutrition in the intensive care unit. Clin Nutr.

[147] Choi EY, Park DA, Park J (2015). Calorie intake of enteral nutrition and clinical outcomes in acutely critically ill patients: a meta-analysis of randomized controlled trial. J Parenter Enteral Nutr..

[148] McClave SA, Taylor BE, Martindale RG, Warren MM, Johnson DR, Braunschweig C (2016). Gudielines for the provision and assessment of nutrition support therapy in the adult critically ill patient: society of Critical Care Medicine (SCCM) and American Society for Parenteral and Enteral Nutrition (A.S.P.E.N). J Parenter Enteral Nutr..

[149] Elke G, Hartl WH, Kreymann KG, Adolph M, Felbinger TW, Graf T (2019). Clinical nutrition in critical care medicine-Guideline of the German Society for Nutrition Medicine(DGEM). Clin Nutr ESPEN..

[150] Zhang JY, Shao CH, Yang JH, Su JG, Qian T, Liu JF, et al. Recommendations for nutrition therapy in critically ill COVID-19 patients. Chin J Clin Med. 2020. http://kns.cnki.net/kcms/detail/31.1794. R.20200311.0958.002.html.

[151] Liew MF, Siow WT, Yau YW, See KC (2020). Safe patient transport for COVID-19. Crit Care.

